# Broodstock nutritional programming differentially affects the hepatic transcriptome and genome-wide DNA methylome of farmed gilthead sea bream (*Sparus aurata*) depending on genetic background

**DOI:** 10.1186/s12864-023-09759-7

**Published:** 2023-11-07

**Authors:** F. Naya-Català, A. Belenguer, D. Montero, S. Torrecillas, B. Soriano, J. Calduch-Giner, C. Llorens, R. Fontanillas, S. Sarih, M. J. Zamorano, M. Izquierdo, J. Pérez-Sánchez

**Affiliations:** 1 Nutrigenomics and Fish Growth Endocrinology Group, Institute of Aquaculture Torre de La Sal (IATS, CSIC), 12595 Castellón, Spain; 2 Grupo de Investigación en Acuicultura (GIA), IU-ECOAQUA, Universidad de Las Palmas de Gran Canaria, Ctra. Taliarte S/N, 35214 Telde, Las Palmas, Canary Islands, Spain; 3 Biotechvana, Parc Científic Universitat de València, 46980 Paterna, Spain; 4 Skretting Aquaculture Research Centre, Stavanger, Norway

**Keywords:** Fish, Nutritional programming, Genetic background, Fish oil, Epigenetics, Transcriptomics, Lipid metabolism, Lipogenesis, DNA-methylation, MBD-seq

## Abstract

**Background:**

Broodstock nutritional programming improves the offspring utilization of plant-based diets in gilthead sea bream through changes in hepatic metabolism. Attention was initially focused on fatty acid desaturases, but it can involve a wide range of processes that remain largely unexplored. How all this can be driven by a different genetic background is hardly underlined, and the present study aimed to assess how broodstock nutrition affects differentially the transcriptome and genome-wide DNA methylome of reference and genetically selected fish within the PROGENSA® selection program.

**Results:**

After the stimulus phase with a low fish oil diet, two offspring subsets of each genetic background received a control or a FUTURE-based diet. This highlighted a different hepatic transcriptome (RNA-seq) and genome-wide DNA methylation (MBD-seq) pattern depending on the genetic background. The number of differentially expressed transcripts following the challenge phase varied from 323 in reference fish to 2,009 in genetically selected fish. The number of discriminant transcripts, and associated enriched functions, were also markedly higher in selected fish. Moreover, correlation analysis depicted a hyper-methylated and down-regulated gene expression state in selected fish with the FUTURE diet, whereas the opposite pattern appeared in reference fish. After filtering for highly represented functions in selected fish, 115 epigenetic markers were retrieved in this group. Among them, lipid metabolism genes (23) were the most reactive following ordering by fold-change in expression, rendering a final list of 10 top markers with a key role on hepatic lipogenesis and fatty acid metabolism (*cd36*, *pitpna*, *cidea*, *fasn*, *g6pd*, *lipt1*, *scd1a*, *acsbg2*, *acsl14*, *acsbg2*).

**Conclusions:**

Gene expression profiles and methylation signatures were dependent on genetic background in our experimental model. Such assumption affected the magnitude, but also the type and direction of change. Thus, the resulting epigenetic clock of reference fish might depict an older phenotype with a lower methylation for the epigenetically responsive genes with a negative methylation-expression pattern. Therefore, epigenetic markers will be specific of each genetic lineage, serving the broodstock programming in our selected fish to prevent and mitigate later in life the risk of hepatic steatosis through changes in hepatic lipogenesis and fatty acid metabolism.

**Supplementary Information:**

The online version contains supplementary material available at 10.1186/s12864-023-09759-7.

## Background

Many studies in humans and animal models have demonstrated that sub-optimal nutrition during pregnancy and neonatal stages induced metabolic changes that can manifest at the tissue, cellular and molecular levels, leading marked physiological consequences for the offspring [1, 2]. Indeed, nutritional stresses act on genes or gene pathways common to most insults (gatekeeper genes), and such knowledge has contributed to prevent diseases in humans [3] or improve performance in sheep [4] or beef cattle [5]. In fish, early nutritional programming also results in developmental adaptations [6–10], including the improved acceptance and utilization of plant-based diets in a typically marine fish such as gilthead sea bream [11–13]. Such nutritional intervention operates among other paths through changes in the offspring hepatic lipid metabolism, being attention initially focused on fatty acid desaturase 2 (Fads2) that shared a clear functional diversification across fish species [14, 15]. Certainly, Fads2 (Δ6-desaturase) catalyses the first and rate limiting step in the biosynthesis of n-3 long-chain polyunsaturated fatty acids (LC-PUFA) to convert α-linolenic acid (ALA, 18:3n-3) into eicosapentaenoic acid (EPA, 20:5n-3). However, the loss of Fads1 (Δ5-desaturase) in marine fish blocks the availability of these animals to elongate and desaturate EPA until docosahexaenoic acid (DHA, 22:6n-3). Alternatively, Fads2 of some marine herbivorous fish is able to produce DHA from EPA through the Δ4 desaturation pathway [16–19]. Such discovery highlighted that the fish biosynthetic capacity of n-3 LC-PUFA not only depends on the dichotomy between freshwater and marine fish species, but also on the trophic level [20].

Altogether, the above findings reinforce the role of Fads2 as a rate-limiting step in the biosynthesis of n-3 LC-PUFA in marine fish [21, 22], and selective breeding for enhanced broodstock *fads2* expression improved the offspring utilization of plant-based diets (limited supply of n3-LC-PUFA) in gilthead sea bream (*Sparus aurata*) [23]. Otherwise, de novo fatty acid biosynthesis (Δ9-pathway) offers the possibility to mitigate the signs of deficiencies in essential fatty acids through the increased production of monounsaturated fatty acids (oleic acid, 18:1n-9) instead of EPA and DHA. Such adaptive feature will contribute to preserve the fatty acid unsaturation index of membrane phospholipids, and juveniles of gilthead sea bream fed semi-synthetic diets formulated to be deficient in n-3 LC-PUFA shared a marked up-regulated expression of the novo hepatic lipogenic genes, elongase 6 (*elovl6*) and stearoyl-coenzyme A desaturase 1a (*scd1a*) [24]. However, the risk of hepatic steatosis cannot be underestimated with exaggerated or poorly regulated hepatic lipogenesis [25], and broodstock nutritional programming with an enriched-ALA diet served to maintain regulated the enhanced expression of *scd1a* in the gilthead sea bream offspring. This thing resulted in a negative correlation between the hepatic *scd1a* expression and the DNA methylation level of several CpG sites of a CG island of the proximal promoter region that contains a PUFA responsive element [24]. All this supports the highly epigenetic regulated expression of *scd1a* in gilthead sea bream, though further research is needed when considering the extent to which such response might involve other processes, directly or indirectly related to lipid metabolism, and more importantly how all this can be driven by a different genetic background.

The aim of this study is to contribute to solve the gap of knowledge for the interplay between epigenetics and genetics in gilthead sea bream, combining massive gene expression analysis (RNA-seq) with genome-wide DNA methylation approaches, for which the gold-standard technique is the whole genome bisulphite sequencing (WGBS). This precise method provides a single-base CpG resolution [26–28], but sequencing to a sufficient coverage can result economically unaffordable. Reduced representation bisulphite sequencing is a cost-effective alternative [29, 30] that has been used in aquaculture for targeting the CG-rich genomic regions of a wide range of species, including European sea bass [31, 32], Atlantic salmon [33], rainbow trout [34], and Nile tilapia [35]. However, bisulphite conversion-based assays fail to differentiate between 5-methylcytosine and other epigenetic modifications [36–38]. Instead, despite not allowing for a single-base CpG resolution, the methyl-binding domain sequencing (MBD-seq) offers an economical alternative for a large coverage of the CpG methylome [39–41] that approximates the sensitivity/specificity of WGBS [42]. Such approach was successfully employed in human and animal studies [43–46], including fish [47, 48] and it was applied herein to underscore how broodstock nutrition with low fish oil feed formulations was affected by selective breeding within the PROGENSA® gilthead sea bream program, which selected for fast growth [49] and a low incidence of skeletal deformities [50], but also for changes in behaviour and swimming performance [51, 52], and intestinal microbiota plasticity to cope with changes in diet composition [53–55].

## Results

### Fish performance

Data of fish performance are shown in Table 1. Initial body weight was similar in all animals, regardless of diet and genotype. Reference (REF) fish presented a significantly lower final body weight (BW) compared to genetically selected (GS) fish (*P* < 0.001). Similarly, diet affected gilthead sea bream weight gain, presenting fish fed FUTURE diet lower final BW than fish fed control (CTRL) diet (*P* < 0.001) within the same genotype. At the end of the experimental period, as shown by the daily growth index (DGI), GS fish fed the CTRL diet grew significantly (*P* < 0.05) better than the animals used for the rest of the treatments, whereas GS fish fed the FUTURE diet performed similarly to those REF fish fed the CTRL diet, and REF fish fed the FUTURE diet presented the lowest (*P* < 0.05) values. Statistical analysis revealed also a significant effect of genotype and diet on specific growth rate (SGR; *P* < 0.001). Additionally, either fish genotype or diet formula affected diet utilization, presenting GS gilthead sea bream better feed conversion ratio (FCR) than the REF animals (*P* < 0.001) as well as fish fed the CTRL diet showed lower FCR than fish fed FUTURE diet (*P* < 0.05).

**Table 1:** Growth and feed utilization parameters in genetically selected fish (GS) and reference fish (REF) fed either FUTURE or CTRL diets during the challenge phase

	**GS**		**REF**		**ANOVA *P* values**		
	CTRL	FUTURE	CTRL	FUTURE	Genotype (G)	Diet (D)	GxD
Initial BW^1^ (g)	12.54 ± 0.2	12.52 ± 0.6	12.5 ± 0.5	12.4 ± 0.3	0.954	0.963	0.921
Final BW^1^ (g)	143.5 ± 5.4	139.9 ± 3.3	133.7 ± 4.1	123.7 ± 7.9	0.0001	0.0001	0.081
SL^2^ (cm)	18.7 ± 0.3	18.2 ± 0.4	18.2 ± 0.3	17.8 ± 0.3	0.621	0.125	0.078
K^3^	2.4 ± 0.1	2.2 ± 0.3	2.1 ± 0.1	2.0 ± 0.1	0.001	0.01	0.067
SGR^4^	1.62 ± 0.04	1.61 ± 0.02	1.58 ± 0.04	1.53 ± 0.05	0.0001	0.0001	0.056
FCR^5^	1.19 ± 0.1	1.17 ± 0.1	1.21 ± 0.2	1.32 ± 0.1	0.0001	0.02	0.061
DGI^6^	1.94 ± 0.1^a^	1.91 ± 0.0^b^	1.86 ± 0.1^b^	1.78 ± 0.1^c^	0.0001	0.02	0.01

Values expressed in mean ± SD. (*n* = 4 tanks/diet/genotype). When the interaction GxD is significant, means bearing different superscript letters differ significantly

^1^Body weight (BW)

^2^Standard length (SL)

^3^Condition factor (K) = [(weight)*100/(length)^3^]

^4^Specific Growth Rate (SGR) = (Ln (final weight)—Ln (initial weight)) * 100/feeding period (days)

^5^Feed Conversion Ratio (FCR) = (total feed fed/total weight gained)

^6^Daily Growth Index (DGI) = [(final weight^1/3^—initial weight^1/3^) * 100 / number of days]

### Patterns of offspring gene expression

**Fig. 1:**
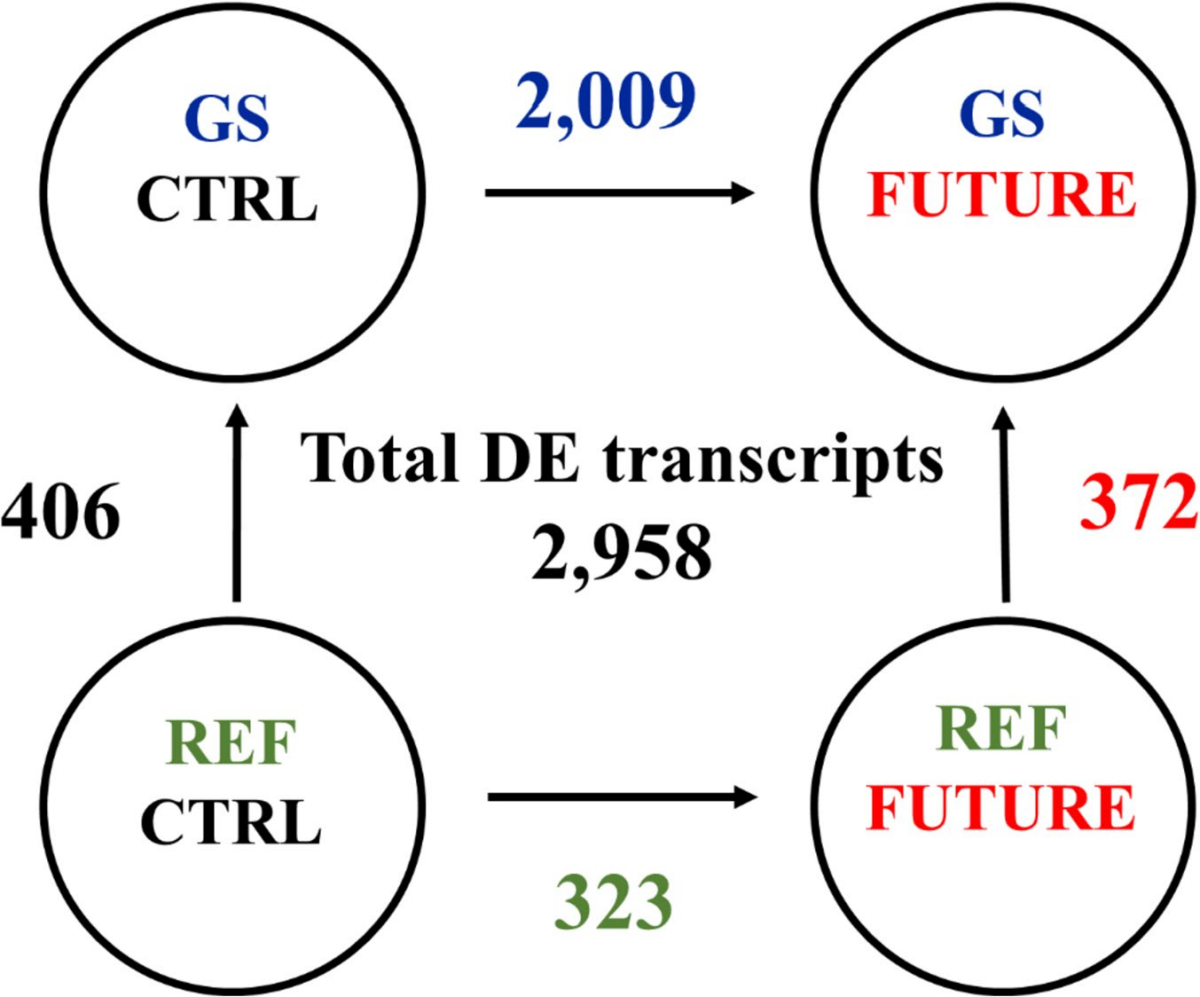
Differentially expressed (DE) transcripts in genetically selected fish (GS) and reference fish (REF) fed either FUTURE or CTRL diets during the challenge phase. Numbers indicate differentially expressed transcripts (FDR-adjusted *P* < 0.05)

Illumina sequencing of offspring mRNA liver samples from crosses of REF or GS fish challenged with a CTRL or FUTURE diet generated ~ 2,008 million paired-end (PE) reads (2 × 150), with an average of ~ 83 million reads per sample (Additional file 1: Supplementary Table 1). After trimming and quality filtering, around 9% of all liver reads were discarded, and the remaining reads ranged between 49 million (~ 7.35 Gb) and 106 million (~ 15.9 Gb) in all samples. Up to 92% of these pre-processed reads were mapped against the IATS-CSIC gilthead sea bream reference genome, which retrieved 46,545 expressed coding transcripts (94.8% of total predicted unique transcripts), corresponding to 20,177 unique descriptions (UD). Differential gene expression analysis resulted in 10,859 transcripts significantly changing among groups when One-way ANOVA (P < 0.05) was applied. The number of differentially expressed (DE) transcripts decreased to 2,958 with FDR-adjusted P < 0.05, being used this set of genes for initial comparisons among groups. Such approach yielded 2,009 DE transcripts (1324 UD) when comparing the diets (FUTURE vs CTRL) within the GS lineage, whereas the same comparison in REF fish only disclosed 323 DE transcripts (207 UD) (Fig. 1). On the other hand, similar results were found when comparing genotypes (GS vs REF) within the CTRL-fed fish (406 DE, 263 UD) or within the animals receiving the FUTURE diet (372 DE, 233 UD). These findings indicate a strong interaction between nutritional programming and genetic background when matching the differential offspring transcriptional response to diet for a given genotype, but not for a given diet and the achieved response with REF and GS fish genotypes. As a validation procedure, six genes covering a wide range of up- and down-regulation between FUTURE and CTRL diets in GS fish were selected, and their fold-change values calculated using real-time PCR were quite consistent (r = 0.998) with those of the RNA-seq analysis (Additional file 2: Supplementary Table 2).

### Discriminant and Gene Ontology analysis

**Fig. 2:**
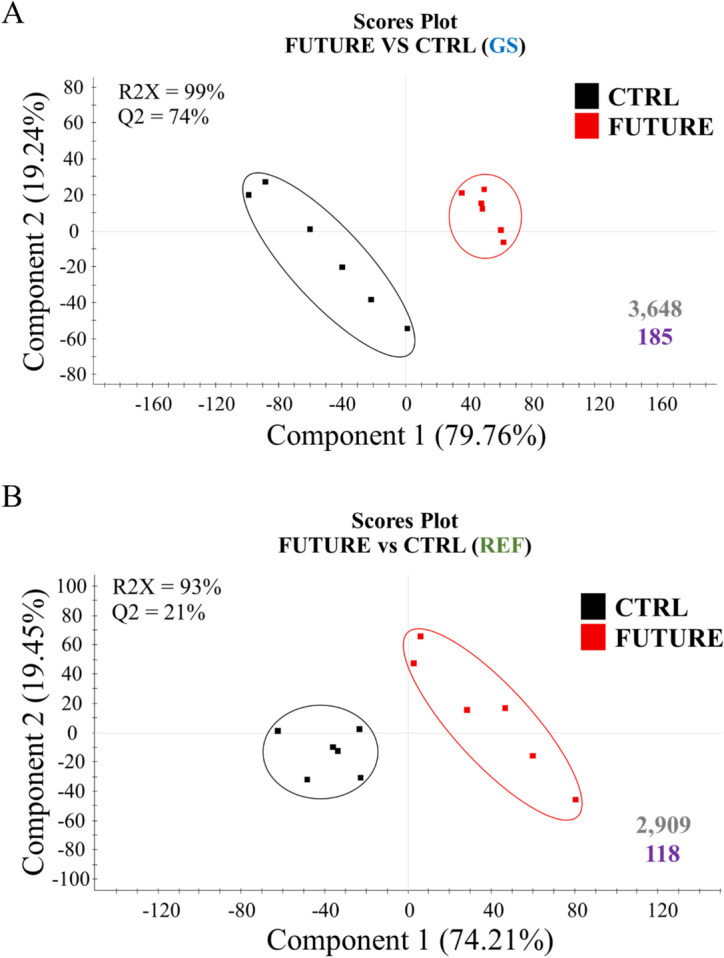
Scores plot of partial least-squares discriminant analysis (PLS-DA) of hepatic transcripts after the challenge phase with CTRL and FUTURE diets in GS (**A**) and REF (**B**) fish. RNA-seq data in the analysis were normalized values of differentially expressed transcripts (One-way ANOVA, *P* < 0.05). The number of discriminant transcripts (VIP ≥ 1; in grey), together with the filtered and enriched functions (GO-BP; in purple) are shown at the bottom right of each plot

To better explore the different nutritionally mediated response of the offspring with a different genetic background, a partial least squares-discriminant analysis (PLS-DA) was conducted with the data filtered by One-Way ANOVA in GS fish fed CTRL or FUTURE diets, and REF fish fed CTRL or FUTURE diets (Fig. 2). The two discriminant models were validated significantly for either GS fish (Additional file 3: Supplementary Fig. 1A) or REF fish (Additional file 3: Supplementary Fig. 1B), being all animals correctly classified in each group by hierarchical cluster analysis (Additional file 3: Supplementary Figs. 1D and E). However, the number of discriminant transcripts (VIP ≥ 1) was greater in GS fish than in REF fish (3,648 *vs* 2,909). Indeed, the explained variance was similar in the two genetic groups of fish (R2X = 93%-99%), whereas the predicted variance was higher in GS fish (Q2 = 74%) than in REF fish (Q2 = 21%) (Fig. 2).

**Fig. 3:**
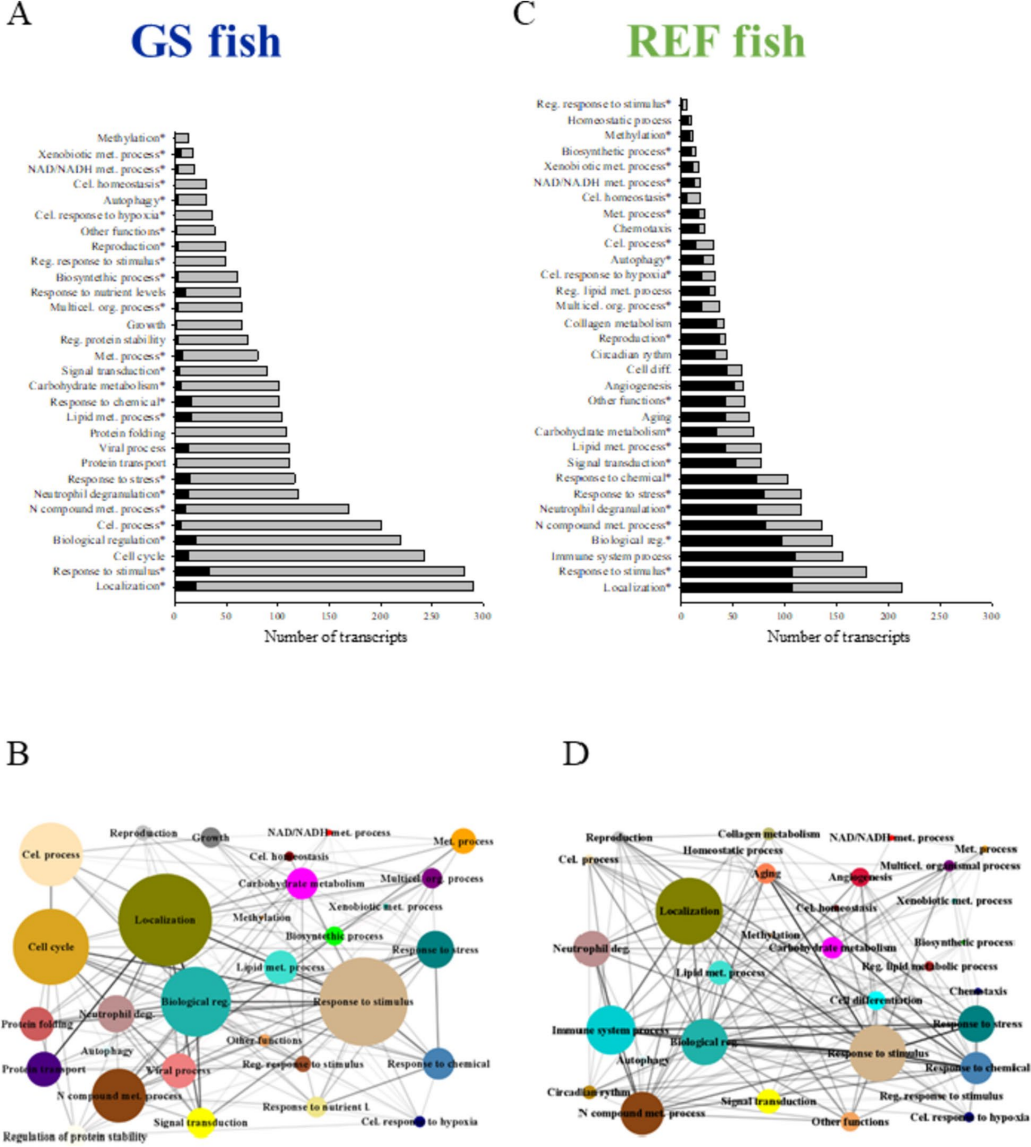
Bar plot depicting the results of an over-representation test performed over the GO-BP terms of the filtered transcripts for the GS (**A**) and REF (**C**) fish. These transcripts were classified in the different GO-BP ancestors presented in the figures. The size of the bars represents the number of transcripts, which are up-regulated (in black) or down-regulated (in grey). Network layout representing the associations between the assigned GO-BP ancestors according to their shared allocated genes in the GS (**B**) and REF (**D**) fish. Node size represents the number of transcripts and node colours, the representative name of GO-BP ancestor. Edge width represents the number of shared genes between two supra-categories. * indicates that the supra-category appears in both GS and REF fish. Met., metabolic; Cel., cellular; Reg., regulation; Multicel. org., multicellular organismal

The over-representation analysis of the list of discriminant transcripts discerned a larger number of enriched functions (Gene ontology—Biological process, GO-BP, unique terms) that was higher in GS fish (546) than in REF fish (242) (Additional file 4: Supplementary Tables 3A and B). For the simplification of the analysis, data were filtered for the enriched functions of upper levels of GO-BP categories, reducing the numbers of terms to 185 in GS fish and 118 in REF fish (Figs. 2A and B). The resulting over-represented functions were clustered in 39 supra-categories (GO-BP ancestors), and the different numbers of DE transcripts within each one are presented in Figs. 3A and C. In both GS and REF fish, the GO-BP supra-categories Localization and Response to stimulus showed the largest number of DE transcripts, although other relevant supra-categories, such as Lipid metabolic process or N compound metabolic process were largely represented. Interestingly, most of these supra-category transcripts (nearly 92%) were down-regulated by FUTURE diet in the offspring of challenged GS fish, whereas the opposite trend occurred in REF fish (nearly 65% of all transcripts were up-regulated). Networks were also performed to show the associations among the different supra-categories of over-represented GO-BP terms, which in turn resulted in an extensive number of links in both GS (Fig. 3B) and REF fish (Fig. 3D).

### Patterns of offspring DNA methylation

Using MBD-seq, ~ 750 million single-end (SE) reads (1 × 75), containing at least one methylated CpG, were obtained, with an average of ~ 31 million reads per sample (Additional file 1: Supplementary Table 1). After trimming and quality filtering, around 6% of all liver reads were discarded, and the remaining reads ranged between 22 million (~ 1.65 Gb) and 40.5 million (~ 3 Gb) in all samples. Up to 84% of these pre-processed reads were mapped against the CSIC gilthead sea bream reference genome (1.6 Gb), which was divided in 25-bp windows (the unit for calculating the level of methylation). Such analysis identified a total of ~ 10 M 25-bp genomic regions that appeared to contain CG dinucleotides susceptible to be altered by methylation. These methylated regions spanned ~ 263 Mb of the total gilthead sea bream genome, and comprised ~ 13.6 M CpG (74.3% of the total 18.3 M CG in the gilthead sea bream genome).

**Fig. 4:**
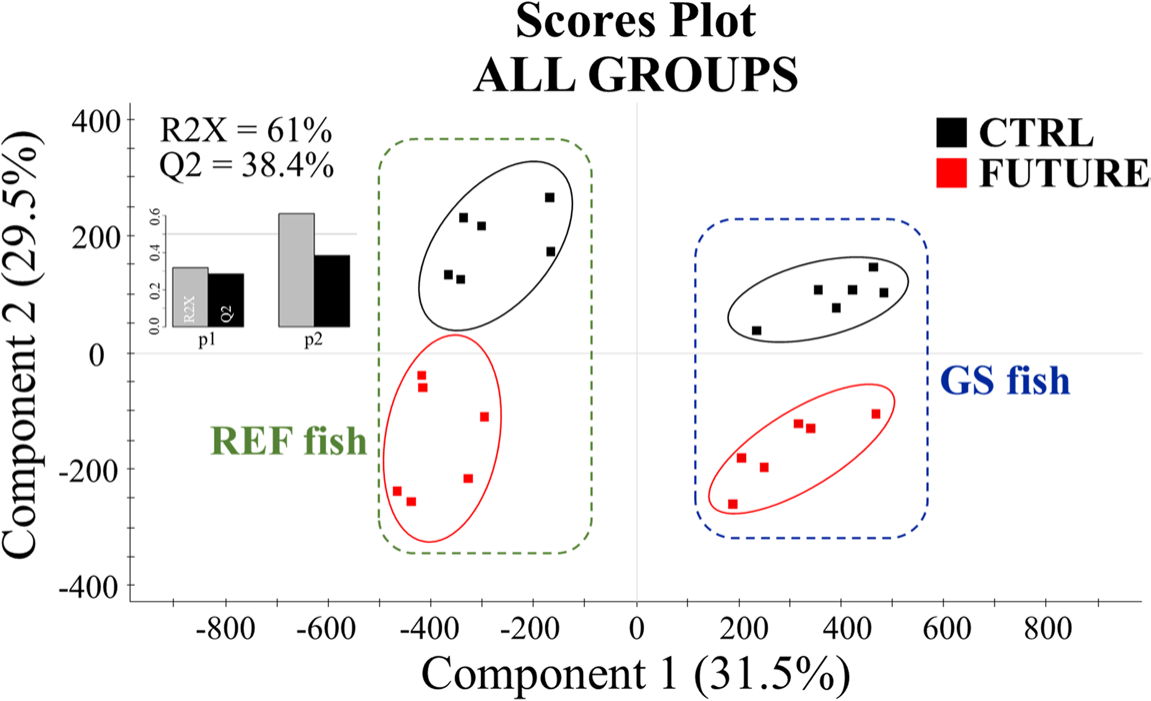
Scores plot of partial least-squares discriminant analysis (PLS-DA) of methylated regions in GS and REF fish fed the FUTURE (in red) or the CTRL (in black) diet after the challenge phase. MBD-seq data were the normalized values of differentially methylated 25-bp genomic regions (One-way ANOVA, *P* < 0.05). Graphical representation of the goodness-of-fit of the PLS-DA model is presented at the upper left of the plot

Differential methylation studies detected a total of ~ 553,000 25-bp genomic regions changing (*P* < 0.05), at least, in one comparison within the four experimental groups. The normalized methylation values (rpkm, reads per kilo base per million mapped reads) of these differentially methylated (DM) regions were used as input in discriminant analysis to assess the effect of nutritional programming and genetic background over DNA-methylation patterns. Unlike RNA-seq results, when both dietary groups were compared within each genetic background, the discriminant separation was not statistically significant (*P* > 0.05). Nonetheless, when the four groups were analysed together, the discriminant model was statistically validated (Additional file 3: Supplementary Fig. 1C), being 21 out of 24 fish correctly classified by hierarchical clustering analyses (Additional file 3: Supplementary Fig. 1F). The resulting model showed percentage values of explained (R2X) and predicted (Q2) variance that remained above 60% and 38%, respectively (Fig. 4). When VIP threshold (VIP ≥ 1) was applied in the validated PLS-DA model, up to 223,154 25-bp DM genomic regions drove the separation between experimental groups. These DM regions of discriminant value were found to be located in the promoter/coding sequence of over 34,000 DM transcripts.

### Differential gene expression and DNA methylation overlapping

**Fig. 5:**
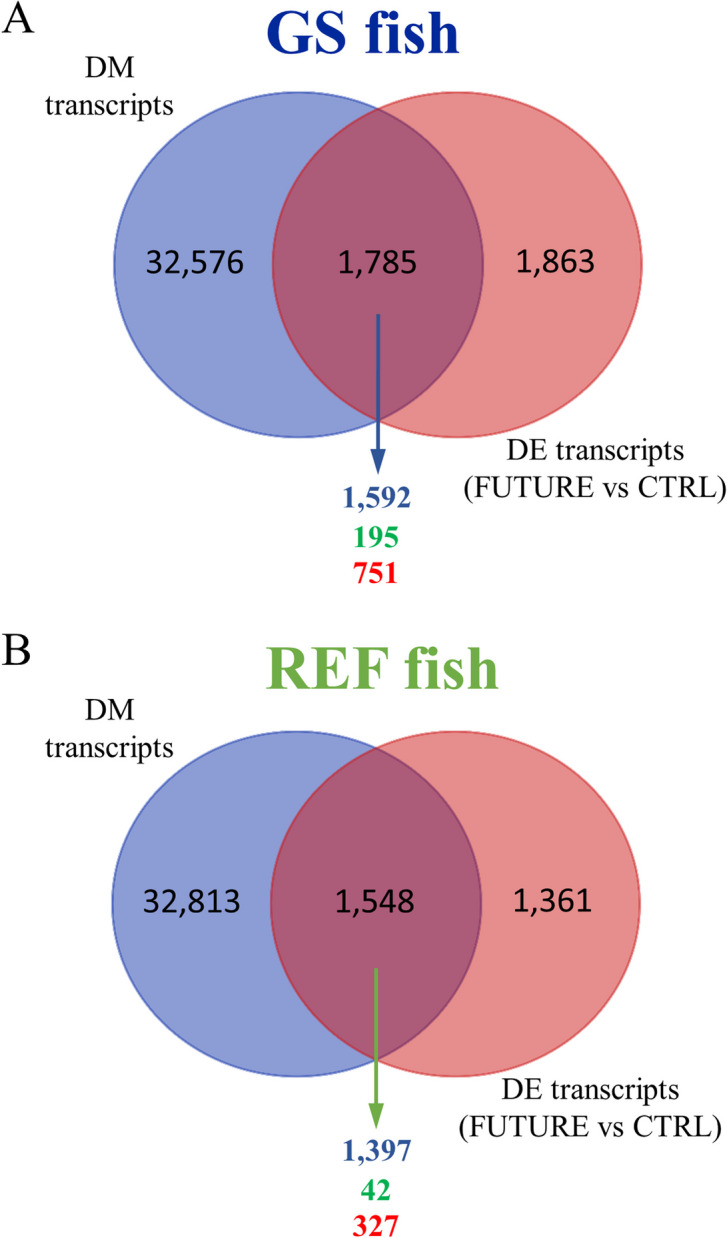
Venn diagrams showing the results of overlapping differentially methylated (DM) transcripts by MBD-seq (One-way ANOVA, *P* < 0.05, VIP ≥ 1) and differentially expressed (DE) transcripts by RNA-seq (One-way ANOVA, *P* < 0.05; VIP ≥ 1) following the challenge phase with FUTURE and CTRL diets in GS (**A**) and REF (**B**) fish. The numbers of unique gene descriptions corresponding to the overlapping genes are in blue. The numbers of enriched functions (GO-BP terms, in green) and their unique gene descriptions (in red) are also presented

**Fig. 6:**
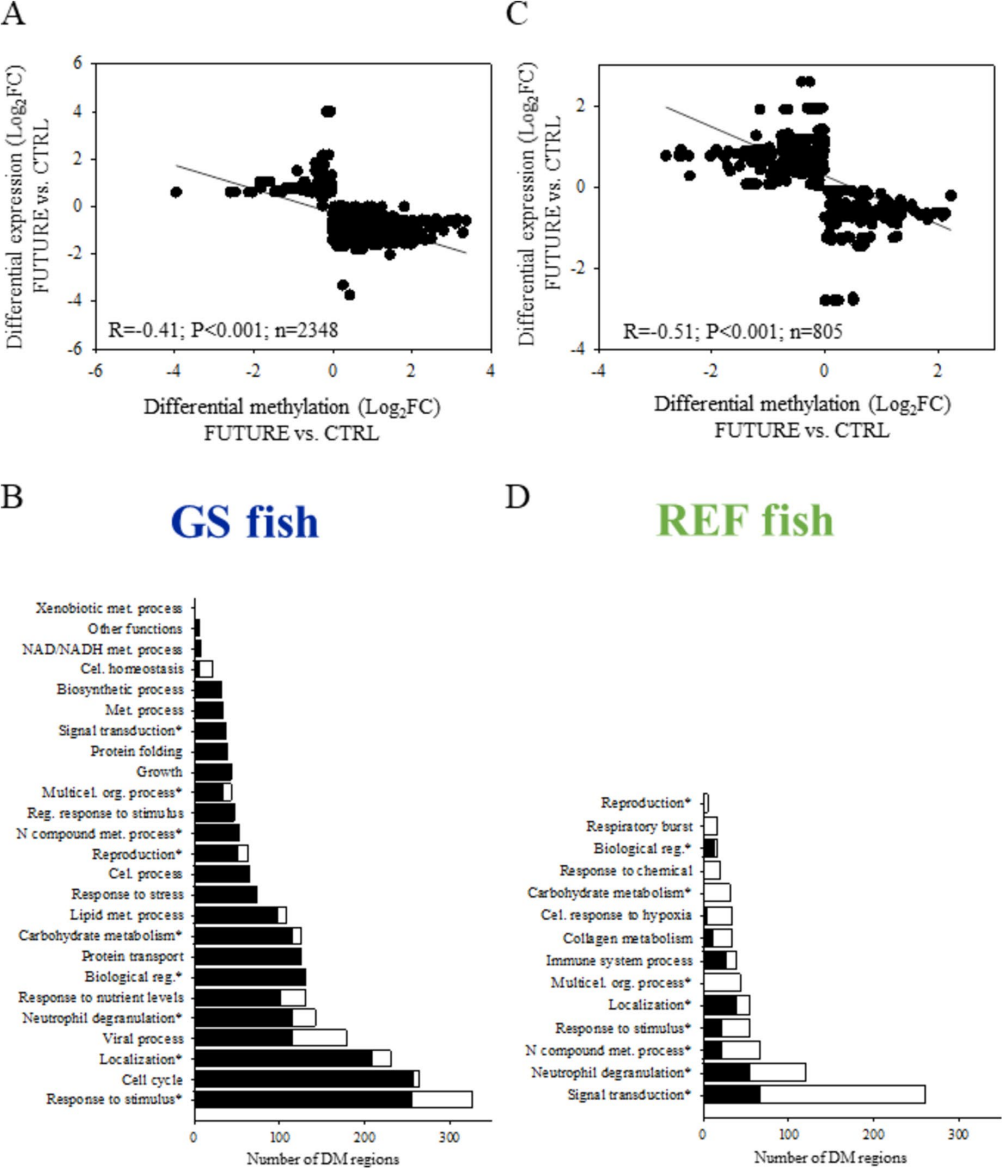
Correlation analysis between differentially methylated (DM) genomic regions and their corresponding transcripts (both as log_2_ fold change in rpkm values between FUTURE and CTRL-fed fish) in GS (**A**) and REF (**C**) fish. Significance of correlation is shown. The results of an over-representation test performed over the GO-BP terms of filtered DM regions showing opposite trends for DNA methylation and the expression of the matching gene are also presented for GS (**B**) and REF (**D**) fish. The GO-BP ancestors assigned to these selected transcripts are presented. The size of the bars represents the number of DM regions, which are hyper-methylated (in black) or hypo-methylated (in white). * indicates that the supra-category appears in both GS and REF fish

When the previously mentioned discriminant DE and DM transcripts were matched, a total of 1,785 (1,592 UD) and 1,548 (1,397 UD) transcripts were overlapping in GS (Fig. 5A) and REF (Fig. 5B) fish, respectively. Functional network analysis of these overlapping genes of fish receiving FUTURE or CTRL diets displayed 195 (854 transcripts; 751 UD; 4,975 DM regions) and 42 (383 transcripts; 327 UD; 2,856 DM regions) different GO-BP terms in GS (Fig. 5A) and REF (Fig. 5B) fish, respectively (Additional file 4: Supplementary Tables 3C and D). Then, we kept with those DM regions that showed an opposite trend for DNA-methylation and expression (i.e., hyper-methylated regions with down-regulation of the matching transcript or vice versa), and after filtering for transcripts with at least 80% of their DM regions with a negative correlation, we displayed a total of 2,348 and 805 DM regions in GS fish (Fig. 6A) and REF fish (Fig. 6C), respectively. Of note, DM regions corresponding to 30 GO-BP ancestors were located in 264 (246 UD) and 99 (96 UD) DE transcripts of GS (Fig. 6B) and REF fish (Fig. 6D), respectively. Most of these DM regions (nearly 88%) were hyper-methylated in GS fish fed FUTURE diet compared to those receiving the CTRL diet, and subsequently their matching genes were down-regulated. Conversely, the opposite pattern occurred in REF fish, where around 66% of all DM regions were hypo-methylated in animals fed FUTURE diet vs those ingesting the CTRL diet. The distinct effect of diet depending on the genetic background seems to indicate again a clear interaction between genetic lineage and nutritional programming. The wide distribution of DM regions across introns (48%), exons (27.4%) and promoters (24.6%) is shown for GS and REF fish in Additional file 5: Supplementary Fig. 2.

### Candidate offspring epigenetic markers

**Fig. 7:**
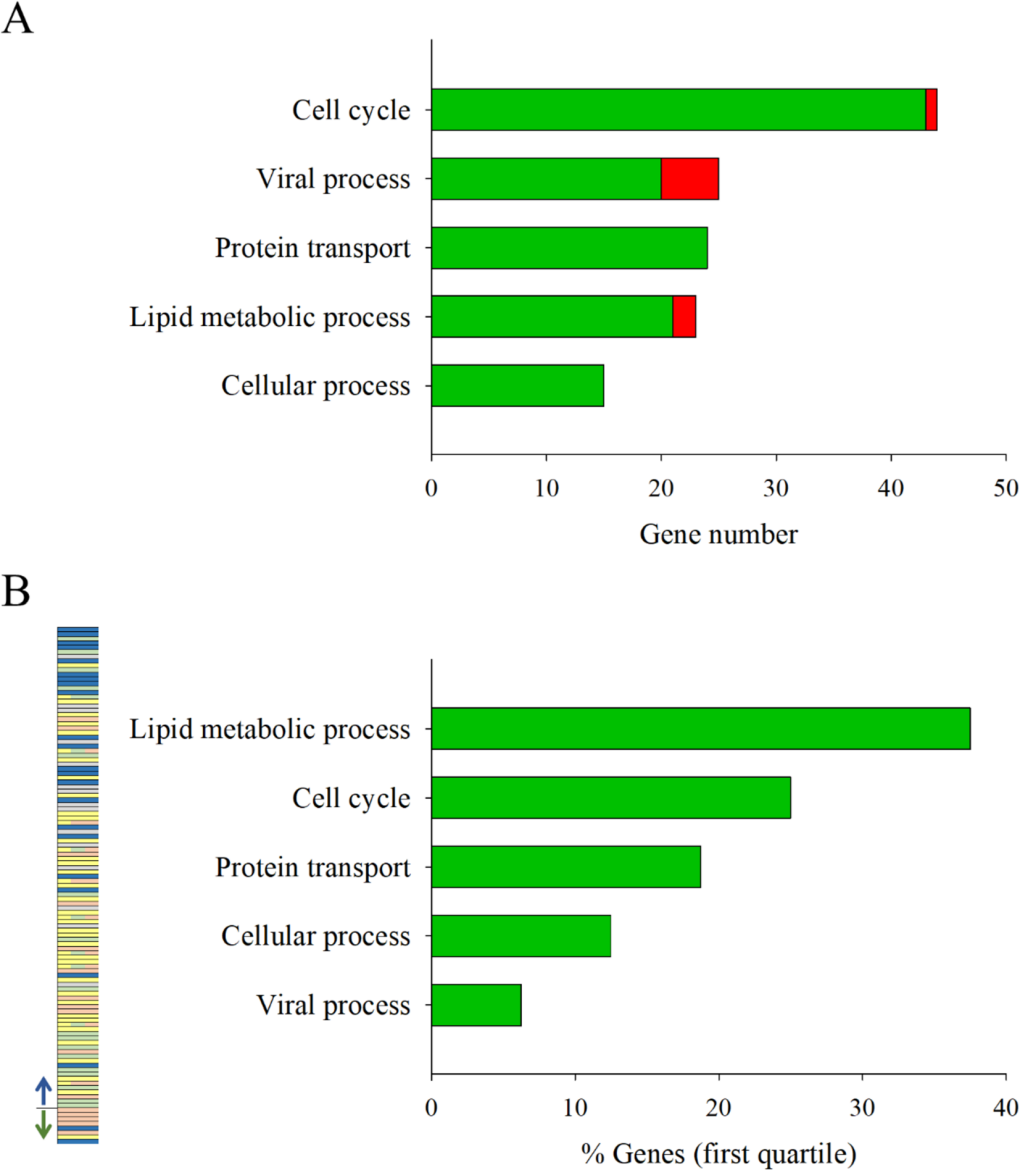
Bar chart representing the top five GO-BP supra-categories of GS fish showing a negative correlation for at least the 80% of differentially methylated regions and their associated differentially expressed transcripts (**A**). Green colour indicates hyper-methylation (FUTURE *vs* CTRL fed fish), whereas red means hypo-methylation. List of all the genes represented in A are ordered by the value of log_2_ fold-change in gene expression, together with a bar chart with the percentage of genes falling within the first quartile of the mentioned list (considering the absolute value of the log_2_ fold-change) (**B**). In the ordered list, each colour represents a different supra-category, being in blue the genes of the supra-category Lipid metabolic process. The down-regulated genes appear above the line on the left (indicated by a blue arrow) and the up-regulated genes below (green arrow)

**Fig. 8:**
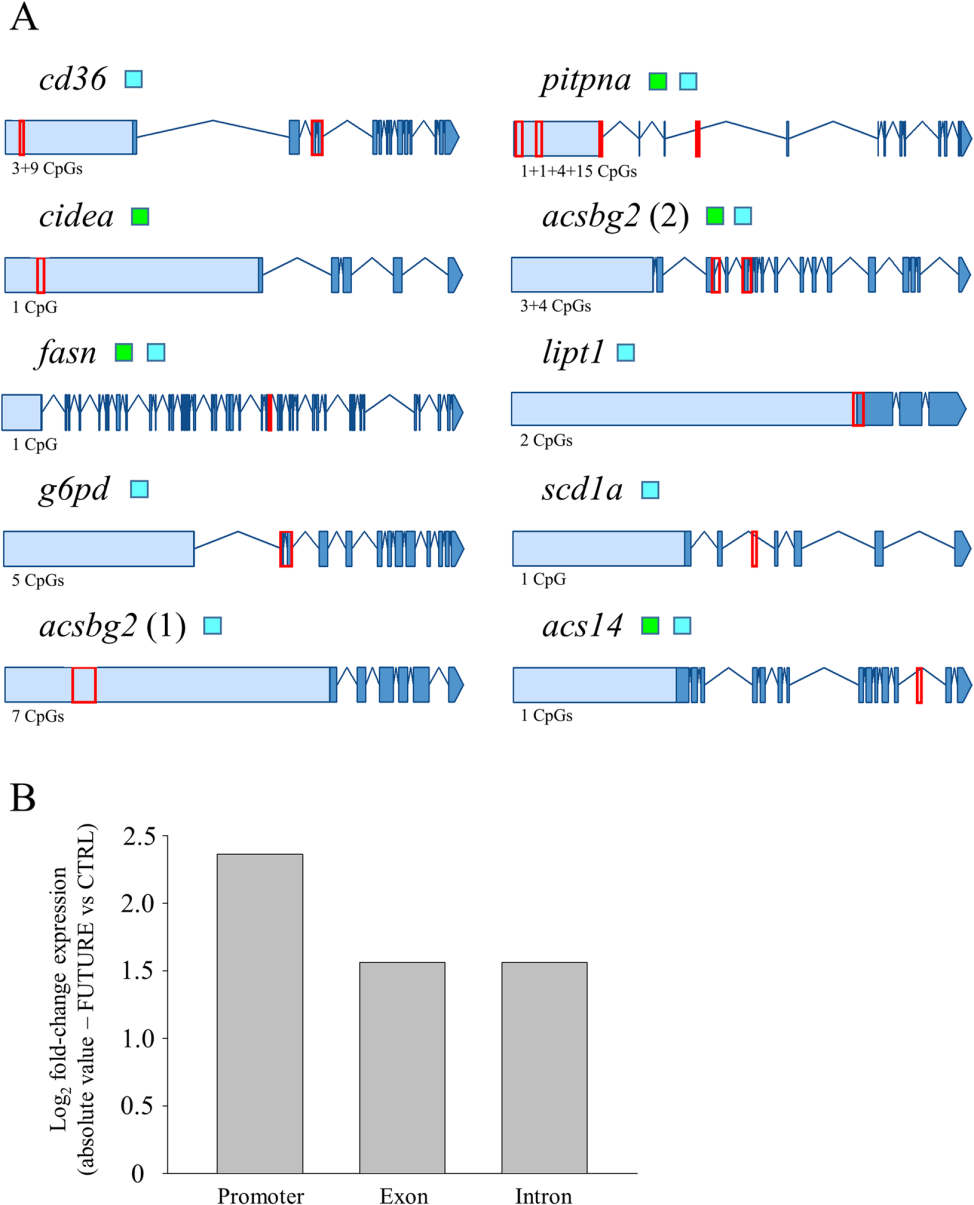
Genomic organization of the 10 top genes belonging to the GO-BP Lipid metabolic process with the greatest absolute change in gene expression during the challenge phase in genetically selected fish (**A**). Red boxes indicate the location of the differentially methylated regions. The number of CpGs within each region is shown below the gene representation and the coloured squares refer to the presence of transcription factor binding sites (green for AP-1 and blue for HNF-3). Bar chart representing the average absolute value of log_2_ fold-change in gene expression (FUTURE vs CTRL) for the genes shown in A whose differentially methylated regions appear in promoter, exon or intron genomic areas (**B**)

Among the 30 selected GO-BP ancestors, we filtered those (11) with over 85% of genes allocated to the enriched GO-BP terms of GS fish (Fig. 5A), and not to the enriched ones of the REF fish (Fig. 5B). For that, the top-five GO-BP ancestors ordered by the number of genes were Cell cycle, Viral process, Protein transport, Lipid metabolic process, and Cellular process (Fig. 7A). These path ancestors allocated 115 transcripts (106 UD) (Additional file 6: Supplementary Table 4), which were further ordered by their expression fold-change when comparing challenged FUTURE and CTRL dietary groups (Fig. 7B). As a result of this, Lipid metabolic process showed the greatest percentage of genes within the first quartile (~ 40%), followed by Cell cycle (~ 25%). The rest of GO-BP ancestors (Protein transport, Cellular process and Viral process) remained below 20%. Regarding the in-depth study of the 23 genes related to Lipid metabolic process, the position of the DM regions and the number of CpGs are presented in Table 2 and Additional file 7: Supplementary Fig. 3. The genomic organization and number of position of DM CpGs was graphically represented for the top-ten genes (*cd36,* fatty acid translocase; *cidea,* cell death-inducing DNA fragmentation factor; *fasn,* fatty acid synthase; *g6pd,* glucose-6-phosphate dehydrogenase; *acsbg2,* long-chain-fatty-acid-CoA ligase; *pitpna,* phosphatidylinositol transfer protein alpha*; acsbg2,* long-chain-fatty-acid-CoA ligase; *lipt1*, lipoyltransferase; *scd1a*; *acsl4*, acyl-CoA synthetase 4) within the category of Lipid metabolic process (Fig. 8A). Within this top list, those transcripts with a DM region spotted in the promoter presented a higher average value of Log_2_ fold-change in their expression (~ 2.5) than those with their DM regions in exon (~ 1.6) or intron (~ 1.5) genomic regions (Fig. 8B). When searched for enriched transcription factor binding sites (TFBS), hepatocyte nuclear factor 3-beta (HNF-3) and transforming growth factor-beta 1 (AP-1) appeared, but none of them were located within the DM regions of any of the 23 genes related to Lipid metabolic processes (Fig. 8A, Additional file 7: Supplementary Fig. 3).

**Table 2:** Differential expression (log_2_ fold-change FUTURE vs CTRL diet, log_2_ FC (exp)) and location (scaffold and chromosome, chr) of the selected 23 genes (potential epigenetic markers) belonging to the supra-category Lipid metabolic process and their corresponding putative differentially methylated (DM) regions. The genomic area (Feature: promoter, Prom, exon or intron) where these DM regions are positioned and their differential methylation (log_2_ fold-change FUTURE vs CTRL diet, log_2_ FC (met)) are also shown

**Gene**	**Log**_**2**_** FC (exp)**	**Scaffold (chr)**	**Position ** **DM region**	**Feature**	**Log**_**2**_** FC (met)**	**Nº CpG**
Platelet glycoprotein 4 (*cd36*)	3.98	2479	51726-51750	Prom	-0.043	1
			51751-51775	Prom	-0.043	2
			63226-63250	Exon	-0.066	1
			63251-63275	Exon	-0.066	3
			63401-63425	Exon	-0.154	1
			63426-63450	Exon	-0.154	1
			63451-63475	Exon	-0.154	1
			63476-63500	Exon	-0.154	1
			63501-63525	Exon	-0.154	1
Cell death activator CIDE-A (*cidea*)	-3.34	2849 (chr1)	106101-106125	Prom	0.262	1
Faty acid synthase (*fasn*)	-2.05	3645 (chr20)	8587626-8587650	Intron	1.452	1
Glucose-6-phosphate 1-dehydrogenase (*g6pd*)	-1.71	3626 (chr7)	5318751-5318775	Exon	0.226	1
			5318776-5318800	Exon	0.127	1
			5318801-5318825	Exon	0.127	1
			5318901-5318925	Intron	0.291	1
			5318926-5318950	Exon / Intron	0.228	1
Long-chain-fatty-acid-CoA ligase ACSBG2-like – 2 (*acsbg2*)	-1.65	3556	126776-126800	Prom	0.710	1
			126926-126950	Prom	0.701	1
			126951-126975	Prom	0.701	1
			126976-127000	Prom	0.701	2
			127001-127025	Prom	0.701	2
Phosphatidylinositol transfer protein alpha isoform (*pitpna*)	-1.44	3450 (chr17)	157751-157775	Exon	1.767	1
			163426-163450	Exon	1.767	1
			166901-166925	Prom	0.053	3
			167076-167100	Prom	0.376	1
			168076-168100	Prom	0.070	2
			168151-168175	Prom	0.070	1
			168176-168200	Prom	0.060	1
			168201-168225	Prom	0.060	3
			168226-168250	Prom	0.060	2
			168251-168275	Prom	0.060	2
			168276-168300	Prom	0.060	3
			168301-168325	Prom	0.017	1
Long-chain-fatty-acid-CoA ligase ACSBG2-like – 1 (*acsbg2*)	-1.40	3556	109101-109125	Exon	1.174	1
			109201-109225	Intron	1.243	1
			109226-109250	Intron	1.243	1
			110201-110225	Exon	1.209	1
			110251-110275	Exon	1.209	1
			110301-110325	Exon /Intron	1.209	1
			110351-110375	Intron	1.209	1
Lipoyltransferase 1, mitocondrial (*lipt1*)	-1.39	3028	167676-167700	Exon	0.315	1
			167701-167725	Exon	0.362	1
Acyl-CoA desaturase (*scd1a*)	-1.33	3325 (chr15)	360376-360400	Intron	0.712	1
Long-chain acyl-CoA synthetase 9, chloroplastic (*acsl4*)	-1.29	3628 (chr18)	450476-450500	Intron	0.588	1
Acetyl-CoA carboxylase 1 (*acac*)	-1.12	3342 (chr23)	36801-36825	Intron	0.360	1
			36876-36900	Intron	0.360	3
			36976-37000	Exon / Intron	0.341	1
			37001-37025	Exon	0.341	2
			37026-37050	Exon	0.341	2
			37076-37100	Exon	0.341	1
			37126-37150	Intron	0.090	3
Methylsterol monooxygenase 1 (*msmo1*)	-1.10	3027	30476-30500	Prom	0.640	1
			30526-30550	Prom	0.640	1
			30601-30625	Prom	0.640	1
			30626-30650	Prom	0.640	1
Hydroxymethylglutaryl-CoA synthase, cytoplasmic (*hmgcs1*)	-1.05	3483 (chr15)	18201-18225	Exon	2.807	1
			18276-18300	Intron	2.807	2
			18301-18325	Intron	2.807	1
ATP-citrate synthase (*acly*)	-0.99	3591 (chr20)	1499501-1499525	Exon	0.148	1
			1499551-1499575	Exon	0.215	3
			1499576-1499600	Exon	0.215	2
			1499601-1499625	Exon	0.139	1
			1499626-1499650	Exon	0.139	2
			1499651-1499675	Exon	0.139	2
			1499776-1499800	Intron	0.645	1
			1499826-1499850	Exon /Intron	0.647	1
			1499851-1499875	Exon	0.706	2
Acetyl-coenzyme A synthetase, cytoplasmic (*acss2*)	-0.99	3644 (chr21)	558951-558975	Intron	0.264	1
			559001-559025	Intron	0.213	1
			559076-559100	Intron	0.134	1
			559101-559125	Intron	0.134	1
			559176-559200	Intron	0.134	1
Phosphatidylinositol transfer protein beta isoform (*pitpnb*)	-0.95	433	2201-2225	Intron	0.171	1
Alkyldihydroxyacetonephosphate synthase, peroxisomal (*agps*)	-0.89	3567 (chr9)	85801-85825	Intron	0.555	2
			85951-85975	Intron	0.555	1
			86026-86050	Intron	0.555	2
			86151-86175	Intron	0.546	1
			86176-86200	Intron	0.546	1
N-alpha-acetyltransferase 40 (*naa40*)	-0.87	3225	556751-556775	Exon / Intron	1.192	2
			556776-556800	Exon	1.192	1
			556801-556825	Exon / Intron	1.192	1
Dolichyldiphosphatase 1 (*dolpp1*)	-0.80	3620	1620326-1620350	Intron	1.685	1
			1620401-1620425	Intron	1.605	1
			1620451-1620475	Intron	1.605	1
			1620476-1620500	Intron	1.605	1
			1620501-1620525	Intron	1.608	1
			1620526-1620550	Intron	1.608	1
Cytochrome b5 (*cyb5*)	-0.79	3635 (chr4)	2932701-2932725	Prom	0.227	1
			2932801-2932825	Prom	0.097	2
Glycerol-3-phosphate acyltransferase 1, mitocondrial (*gpam*)	-0.64	3591 (chr20)	1271726-1271750	Intron	0.870	1
			1271751-1271775	Exon / Intron	0.870	1
			1271776-1271800	Exon	0.870	2
			1271826-1271850	Exon	0.870	1
			1282876-1282900	Intron	1.253	1
			1282901-1282925	Intron	1.253	1
			1282951-1282975	Intron	1.253	1
Peroxisome proliferator-activated receptor Alpha (*ppara*)	0.62	3594 (chr14)	2131726-2131750	Prom	-0.360	2
			2151576-2151600	Intron	-0.405	1
Microsomal glutathione S-transferase 3 (*mgst3*)	-0.51	3644 (chr21)	7756501-7756525	Prom	0.524	2
			7756526-7756550	Prom	0.475	3

## Discussion

Evidences showing a link between maternal and early life nutrition and epigenetics have been shown and reviewed by several authors in farmed fish [56, 57]. Hence, the transcriptional impact of epigenetic modifications has an effect on the organism's phenotype that results in developmental adaptations that permanently change the structure, physiology and metabolism of affected animals during adult life [58]. Thus, it is well recognized that environmental regulation of economically important aquaculture traits (e.g., growth, disease resistance, low oxygen and warmer temperature tolerance, etc.) is mediated, at least in part, at the level of epigenetic regulation, and such environment-induced epigenetic changes appeared to be intergenerationally inherited, though evidences for transgenerational inheritance are still limited [59]. The fine interplay of genetics and epigenetics is thereby hardly underlying, though a recent study highlighted that the global hepatic methylome landscape and the expression level of DNA (de)methylation-related genes were differentially regulated in trout isogenic lines [60]. In the same line, we found herein that nutritional programming differentially affects the hepatic transcriptome and genome-wide DNA methylome of farmed gilthead sea bream depending on genetic background. Certainly, the offspring of GS fish within the PROGENSA® breeding program shared a better performance than those of REF animals during the challenge phase, and differences observed between fish fed with the CTRL or FUTURE-based diet seemed to be lower in GS lineage. This nutritional challenge was in turn associated with a great impact on the number of genes and functions potentially altered by epigenetic mechanisms, especially in GS animals. Such statement about the latter fish was especially evident for the GO-BP term Lipid metabolic process after the application of different filters that ultimately reflected in a consistent manner the offspring epigenetic-mediated changes on hepatic lipogenesis and fatty acid metabolism. This, together with the observation that, during the challenging phase, the number of DE genes and the associated GO-BP terms was larger in GS fish than in REF fish highly supported an improved metabolic plasticity of GS lineage (Figs. 1, 2 and 3), which was first evidenced herein by a wide-liver transcriptome response. Likewise, previous microbiota studies also supported an improved resilience and functional plasticity of GS fish that can become especially relevant in a scenario of climate change. Certainly, using metatranscriptomic and inferred-metagenome approaches, we found that the adherent intestinal microbiota of GS fish may change their function and activity instead of their composition to cope with changes in diet composition. Such statement was first evidenced at harvest with the offspring of F1/F2 crosses farmed in a Mediterranean experimental facility [53, 54], and thereafter this has been confirmed on a temporal basis through the production cycle of F3/F4 crosses at Canary Islands [55].

Concerning DNA methylation, a large body of evidence in a wide range of fish links changes in epigenetic signatures with a different genetic background [59, 61, 62]. This can also apply to gilthead sea bream and accordingly, in the present study, important differences in the methylation patterns were found when comparisons were made between GS and REF fish (Fig. 4). Moreover, when DM regions were matched with DE transcripts, a remarkable number of DM regions corresponding to DE transcripts were found in response to nutritional programming in either GS or REF animals (Fig. 5), which is in agreement with previous studies in the same [24, 63] or other fish species [8, 10, 64]. However, the number of GO-BP functions associated to DE transcripts whose expression could be potentially altered by different DNA methylation patterns was over 4-fold larger in GS than in REF fish (Fig. 5), which might also imply a greater functional plasticity of the GS linage in terms of epigenetic regulatory mechanisms.

Methylated CpGs are usually associated with the silencing of gene expression [65], so we retained those transcripts showing negative correlation between DNA methylation and their expression level in FUTURE *vs.* CTRL-fed animals within each fish lineage (Fig. 6). Interestingly, most of these DM regions (approximately 88% of all) were hyper-methylated in GS fish receiving the FUTURE diet, whereas in REF animals most of them (approximately 66% of all) were hypo-methylated. These percentages of hyper and hypo-methylation were similar to those calculated for down (almost 92%) or up-regulated (nearly 65%) DE transcripts in the offspring of GS and REF fish during the challenge phase (Fig. 3). Such analogy further supports the relevance of DNA methylation in the regulation of gene expression in fish and in our experimental model in particular [9]. Otherwise, like in humans and other animal models [66, 67], a global DNA hypo-methylation and site-specific hyper-methylation could be associated with aging in marine fish [68, 69]. We may then speculate that changes in the epigenetic clock of REF animals might occur, reflecting perhaps an aging phenotype with an overall impairment of performance that affects among other traits, growth, swimming behaviour and the incidence of skeletal deformities [49–52]. All this reinforces the role of differentially DNA methylation patterns as key epigenetic markers, and after applying stringent filters focused on the differential response of GS fish, up to 23 genes with the GO-BP term Lipid metabolic process were retained as powerful epigenetic markers of early nutritional programming of hepatic metabolism with FUTURE-based diets. An initial prospect of DM regions revealed their wide distribution across the genome, but it is usually believed that differentially methylated CpG sites in the promoter region exert a stronger regulatory effect upon gene expression [70]. Accordingly, when responsive genes related to lipid metabolism were ordered by fold-change in expression, the top 10 genes showed a higher concentration of differentially methylated CpG sites in their promoter region (Fig. 8B). Nonetheless, it should be born in mind that the relationship of the epigenetic modifications with gene expression seems to be far more complex than initially thought [71], and regulatory DM regions in areas different to promoters have been reported across all vertebrate species [31, 72, 73].

Regarding in-depth the list of the top 10 candidate epigenetic markers, *scd1a* was among the selected genes. This gene encodes for the stearoyl-CoA desaturase, which has a role in the synthesis of 18C and 16C monounsaturated fatty acids (oleic acid and palmitoleic acid) from stearoyl-CoA and palmitoyl-CoA, respectively [74]. In a previous gilthead sea bream study, a reduced expression of *scd1a* was concurrent with a higher DNA methylation level in the gilthead sea bream offspring of parents fed an ALA-enriched diet with a limited supply of n-3 LC-PUFA [24]. In particular, that regulation was associated to an increased methylation at a regulatory region in the proximal *scd1a* promoter, a genomic area rich in hypo-methylated CGI [75]. Conversely, in the present study a differentially methylated cytosine was found in the second intron instead of the promoter region (Fig. 8A). It can be argued that fish and experimental conditions are not the same in this and the previous study, but importantly Perera et al. [24] focused on CGI of the proximal promoter that mostly becomes hypo-methylated, especially in the case of genes with a constitutive high expression level [76]. By contrast, we used herein a wide-genome approach (MBD-seq) that primes the recognition of differentially methylated CpG sites on methylated genomic regions [41]. In this regard, the no coincidence of DM regions in this and the previous [24] study could be mainly attributed to the complementarity of methodologies rather than to the discordance of results.

Besides *scd1a*, other candidate epigenetic markers are the *g6pd* gene that encodes for the glucose-6-phosphate dehydrogenase, a major regulatory enzyme involved in the generation of NADPH, which is required by the *fasn* for catalysing all the reaction steps involved in the conversion of acetyl-CoA and malonyl-CoA to palmitate [77, 78]. Other important lipogenic enzyme is *lipt1, a* lipoyltransferase that is required for 2-ketoacid dehydrogenase function and mitochondrial fatty acid synthesis [79]. The relative contribution of all these genes, in addition to that of *scd1a*, on the resulting lipid metabolism phenotype remains elusive, but all of them showed significantly hyper-methylated CpG sites with a concurrent down-regulated expression in the offspring of GS fish fed the FUTURE diet. Similar DNA-methylation and gene expression patterns were found for *cidea* and *pitpna*. The former is a key landmark of apoptosis that plays a crucial role in lipid and energy metabolism including lipolysis, lipid oxidation and lipid droplet formation, resulting its low expression in a reduced accumulation of lipid depots [80]. Likewise, the *pitpna* gene encodes for a lipid-binding protein that catalyzes the transfer of phosphatidylinositol and phosphatidylcholine from the Golgi apparatus to the endoplasmic reticulum [81], and its up-regulated expression in zebrafish is concurrent with signs of hepatic steatosis [82]. Overall, this gene expression and epigenetic down-regulated pattern would drive the hepatic lipid metabolism towards reduced hepatic fatty acid biosynthesis and lipid storage, which will act limiting the lipogenic pathway in response to a low fish oil diet during the challenge phase [24]. The fact that this outcome appeared in GS animals is consistent with a recent study where the expression of several hepatic gene markers of lipid metabolism, including *scd1a*, were down-regulated in gilthead sea bream differentially selected for improved feed conversion ratio, suggesting that more efficient fish are also likely to present lowered hepatic lipogenesis and fat deposition rates [83].

Acyl-CoA synthetases, encoded by *acsl4* and *acsbg2* genes, were also identified as robust epigenetic markers of nutritional programming in our model of GS fish. These genes encode for enzymes catalysing the conversion of long-chain fatty acids to their active form acyl-CoA for both synthesis of cellular lipids and degradation via β-oxidation. The Acsl4 enzyme shows preference for arachidonic acid and EPA as substrates [84], whereas the latter has increased ability for oleic acid and linoleic acid [85]. We may then speculate that, in our experimental conditions, with a low fish oil diet, the enhanced DNA methylation and down-regulated expression of *acsl4* and *acsbg2* genes will serve to limit and preserve the use of unsaturated fatty acids for vital functions of the organism. This would be consistent with the idea of an enhanced mobilization of LC-PUFA from liver to other tissues in response to similar attempts of nutritional programming in the same species [23]. Lastly, among the 10 top selected genes, the only one showing hypo-methylation and up-regulation (FUTURE vs CTRL) was *cd36*, whose encoded protein is an important fatty acid transporter associated with long-chain fatty acid trans-membrane uptake [86–88]. This would favour the uptake of available fatty acids, which would be compatible with the described down-regulation of genes related to lipogenesis and hepatic fat storage. Accordingly, Turkmen et al. [13] allowed that early nutritional programming with linseed oil-rich diets reduced the risk of hepatic steatosis in gilthead sea bream. Overall, all this agrees with the initial statement that the epigenetic regulation of gene expression due to nutritional programming works towards a balanced physiological response of the animals by means of precluding an over-expression of specific genes that might result counterproductive in a changing environment [24]. Likewise, experimental evidences in salmon indicated that this general view not only applies to nutritional programming, but also to DNA methylation dynamics of fish challenged with high temperature and moderate hypoxia in a context of global warm [76]. Whether those induced epigenetic marks (mediating the response to nutritional programming) might be transmitted over generations cannot be assessed in this study and further research about their transgenerational inheritance needs to be addressed for its application in aquaculture practice.

## Conclusions

**Fig. 9:**
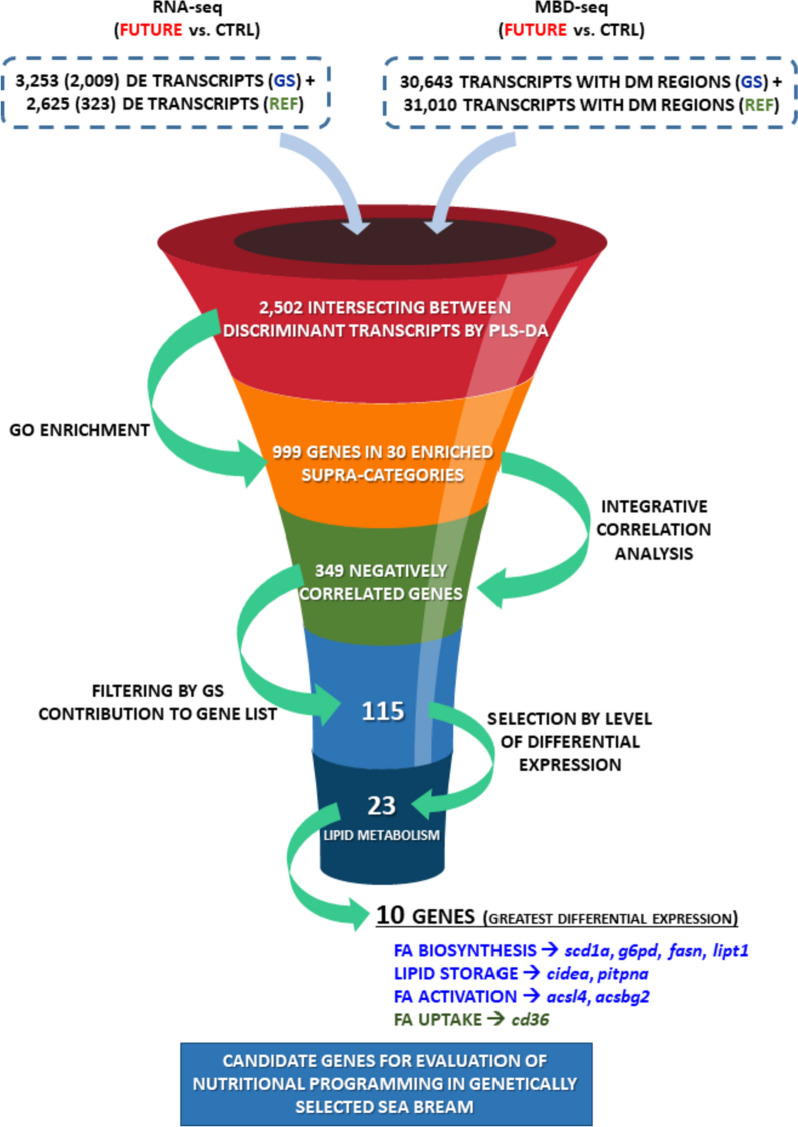
Chart representing the filters and criteria applied for matching wide-transcriptomics (RNA-seq) and wide-genomic DNA methylation (MBD-seq) approaches for recording robust epigenetic markers of special relevance to evaluate the success of nutritional programming in genetically selected (GS) gilthead sea bream within the PROGENSA® program. For the 10 top genes identified as candidate markers, blue colour indicates down-regulation in GS fish challenged with the FUTURE diet, whereas green refers to up-regulation

Gene expression profiles and DNA methylation signatures following nutritional programming were clearly dependent on genetic background in our experimental model. Such assumption affected the magnitude, but also the type and direction of change when comparing GS and REF fish. Accordingly, the resulting epigenetic clock of REF fish might depict an older phenotype with a lower DNA methylation state of DE transcripts during the challenging phase with the FUTURE-based diet formulation. Therefore, attempts to search and validate robust epigenetic markers will be specific of each lineage, and focusing on GS fish we successively applied different filters summarized in Fig. 9, which allows us to reduce the search to 115 candidate epigenetic markers. However, genes with the GO-BP term Lipid metabolism were markedly reordered after filtering by fold-changes in expression. This rendered a final list of top 10 epigenetic markers ordered by the magnitude of response (*cd36* > *cidea* > *fasn* > *g6pd* > *acbsg2* > *pitpna* > *acsbg2* > *lipt1* > *scd1a* > *acsl4*), which reinforces the key role of maintaining regulated hepatic lipogenesis and fatty acid metabolism when precluding the over-expression of specific genes that might result counterproductive in a changing environment.

## Methods

### Ethics statement

All procedures of animal rearing and sampling were carried out according to European animal directives (2010/63/EU) and Spanish laws (Royal Decree RD53/2013) for the protection of animals used in scientific experiments. The Bioethical Committee of the University of Las Palmas de Gran Canaria approved all the protocols used in the present study (approval no. OEBA_ULPGC_26/2019).

### Broodstock crosses

A population of 6,122 adult fish from the Canary Islands at the 3^rd^ generation of National Breeding Program (PROGENSA®) were evaluated for growth. The estimated breeding values (EBV, expressed as g of whole body) ranged between -159.14 for reference fish (REF) and + 223.18 for the selected fish (GS) with an average value of 8.59 and a standard deviation value of 52.84. A subset of 196 fish (98 fish per broodstock) was then selected as breeders with values for the EBV varying from -25.95 in the group of REF fish to + 39.68 in the group of GS fish, comprising almost the 47% of the evaluated population.

### Diets and experimental design

**Fig. 10:**
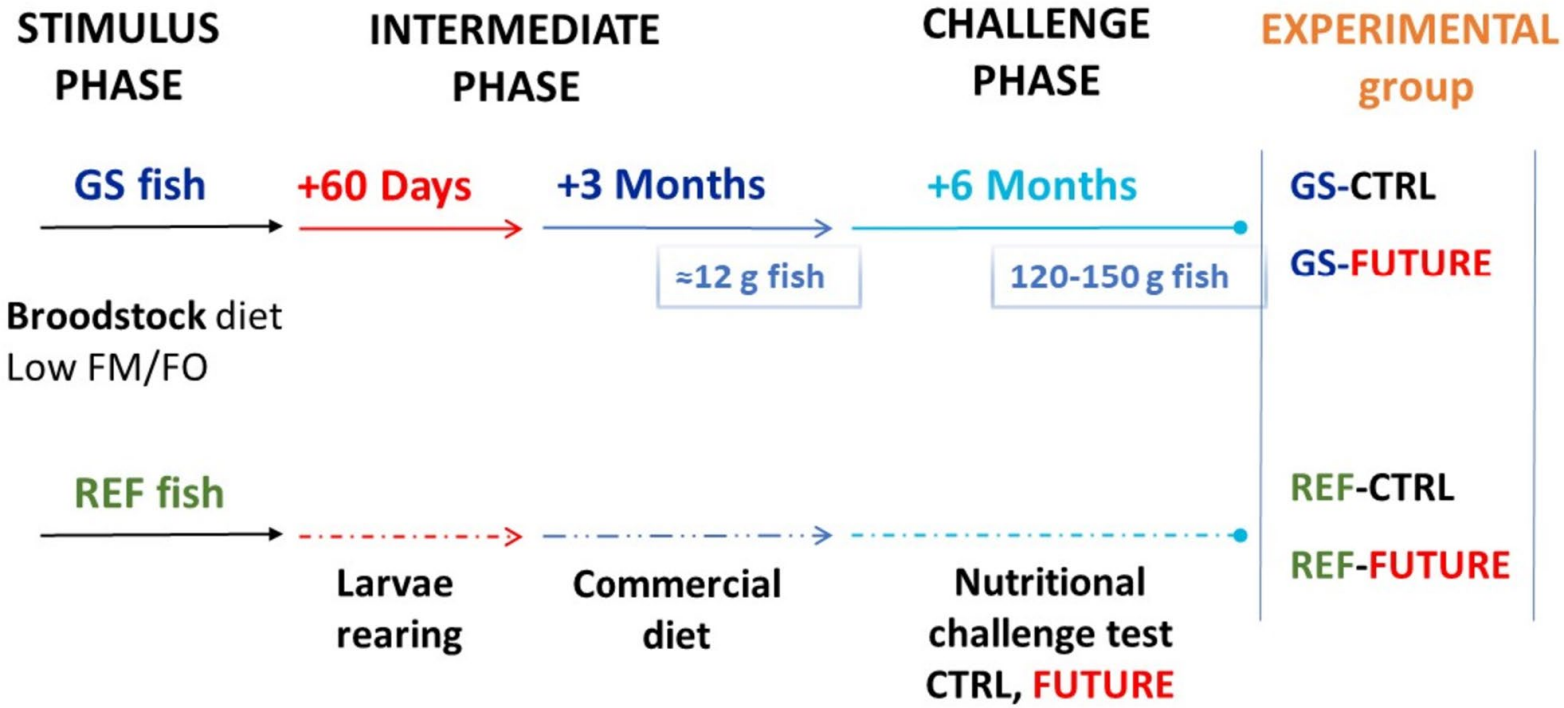
Diagram of the experimental design

Two experimental, with a varying granule size and composition, and the broodstock diets were employed in this study. The control diet (CTRL) contained fish meal (15%) as the main protein source. The alternative diet (FUTURE) was half-reduced in fish meal (7.5%). Fish oil was added at a relatively high level (5.7–7.6%) in CTRL diet, whereas the FUTURE diet was completely devoid of fish oil. Ingredients and composition are presented in Tables 3 and 4. Gilthead sea bream brood fish were used in this study, belonging to fish genetically selected for high growth (GS) or to Reference fish (REF) within the PROGENSA® selection program. Briefly, both groups, GS and REF, were kept in 1,000 L tanks in a flow-through system with filtered seawater, strong aeration, natural photoperiod and temperature conditions in Canary Islands latitude (27º 59’ N; 15º 22’ W) in the experimental facilities of IU-ECOAQUA (University of Las Palmas de Gran Canaria, Spain). All broodstock animals were fed a low fish oil diet for several months prior to the spawning season (stimulus phase; Fig. 10). Ingredients and composition of the broodstock diet are given in Additional file 8: Supplementary Table 5. Eggs were collected at spawning from the two broodstocks and the offsprings were reared under standard conditions for approximately 5 months (intermediate phase). For further details on the broodstock diet and the spawning quality please see Ferosekhan et al. [89]. Then, 1,600 fish (approximately 12 g weight) from the two broodstock groups (800 fish per genotype) were distributed in 24 500 L tanks and fed (6 days per week) either the FUTURE or the CTRL diet twice a day (08:00 and 14:00 h) for approximately 6 months (challenge phase; Fig. 10). At the end of the trial, 24 h-fasted juvenile fish (6 per diet) were anaesthetized using an overdose of natural clove oil. Livers were rapidly excised, frozen in liquid nitrogen and stored at − 80 °C until RNA and DNA extraction for analyses of gene expression and DNA methylation, respectively.

**Table 3:** Ingredients and proximate composition of the experimental diets (control, CTRL, and FUTURE) with different pellet sizes (1.8 and 3 mm)

Ingredients (%):	CTRL-1.8	FUTURE-1.8	CTRL-3	FUTURE-3
Corn gluten	7.95	4	5	5
Hi Pro Soy bean meal^a^	6.5	9.29	6	5.08
Wheat gluten	17.86	18.34	14.90	14.44
Faba bean dehulled^b^	8	8	8	8
Wheat	12.0	11.04	19.01	19.0
Soy protein concentrate^c^	20	20	17	17
Fish oil^d^	5.75		6.71	
Fish meal^e^	15	7.5	15	7.5
Rapeseed oil	3.79	4.65	5.16	6.52
Phosphate	0.75	0.35	0.82	0.44
Vitamin & mineral mix^f^	0.3	0.3	0.3	0.3
Poultry meal^g^		10		10
Poultry oil^h^		2.21		2.1
DHA oil^i^		2.22		2.53
Lecithin	2	2	2	2
**Proximate composition**				
Dry matter	93.0	93.0	93.0	93.0
Moisture	7.0	7.0	7.0	7.0
Crude protein	48.3	48.7	43.0	43.5
Crude fat	16.0	16.0	18.0	18.0
Ash	5.5	5.5	5.3	5.3

Yttrium premix: 0.1%

^a^Soya bean meal: CJ Selecta S.A (Brasil)

^b^Faba beans: Cefetra BV (The Netherlands)

^c^Soya protein concentrate: CJ Selecta S.A (Brasil)

^d^Fish oil: Copeinca, S. A. (Perú)

^e^Fish meal: Norsildmel AS (Norway)

^f^Mineral and Vitamin premix: Trouw Nutrition (The Netherlands)

^g^Poultry meal: Sonac (Belgium)

^h^Poultry oil: Sonac (Belgium)

^i^DHA: Veramaris (Evonik)

**Table 4:** Fatty acid composition (% of Total Fatty Acids—TFA) of the experimental diets (control, CTRL, and FUTURE)

**Fatty acid**	**CTRL**	**FUTURE**	**Fatty acid**	**CTRL**	**FUTURE**
**14:0**	2.67	2.27	**20:0**	0.48	0.43
**14:1n-5**	n.d	0.03	**20:1n-11**	0.30	0.33
**14:1n-7**	0.07	0.06	**20:1n-9**	3.93	3.32
**15:0**	0.22	0.22	**20:1n-7**	0.10	0.08
**16:0**	10.99	10.97	**20:2n-6**	0.22	0.19
**16:1n-7**	2.52	2.21	**20:3n-9**	n.d	0.02
**16:2n-4**	0.29	0.20	**20:3n-6**	0.04	0.03
**16:4n-1**	0.36	0.22	**20:4n-6**	0.14	0.15
**18:0**	2.25	1.99	**20:3n-3**	0.07	0.06
**18:1n-9**	32.94	35.02	**20:4n-3**	0.21	0.20
**18:1n-7**	2.34	2.44	**20:5n-3**	3.91	3.77
**18:1n-5**	0.11	0.12	**22:0**	0.62	0.26
**18:2n-6**	16.84	17.97	**22:1n-11**	5.44	4.65
**18:2n-4**	0.05	0.05	**22:1n-9**	0.96	0.42
**18: 3n-6**	0.04	0.03	**22:1n-7**	0.06	0.05
**18:3n-3**	4.93	5.27	**22:5n-3**	0.33	0.32
**18:4n-3**	1.56	1.26	**22:5n-6**	0.07	0.07
**18:4n-1**	0.05	0.04	**22:6n-3**	4.33	4.71
	**CTRL**		**FUTURE**		
**∑ Saturated**	17.47		16.32		
**∑ Monounsaturated**	49.13		49.11		
**∑ n-3**	15.46		15.71		
**∑ n-6**	17.20		18.35		
**∑ n-3 PUFA**	8.96		9.18		
**n-3/n-6**	0.90		0.86		

*n.d.* not determined, *PUFA* polyunsaturated fatty acids

### DNA and RNA Extraction

Liver DNA was extracted using the Quick-DNA™ Mini-prep Plus Kit (Zymo Research, Irvine, CA, USA) following the manufacturer’s instructions. The quantity and quality of DNA were assessed by a NanoDrop 2000c Spectrophotometer (Thermo Fisher Scientific, Waltham, MA, USA), and DNA integrity was assessed in 1% agarose gels. Total RNA (70–100 μg) from liver was extracted with the MagMAX™-96 Total RNA Isolation Kit (Applied Biosystems, Foster City, CA, United States). The RNA concentration and purity was determined using a NanoDrop 2000c Spectrophotometer (Thermo Fisher Scientific, Waltham, MA, USA). Quality and integrity of the isolated RNA were checked on an Agilent Bioanalyzer 2100 total RNA Nano series II chip (Agilent, Amstelveen, Netherlands), yielding RNA integrity numbers (RINs) between 8 and 10. Samples were stored at -80 ºC until DNA and RNA sequencing.

### DNA and RNA Illumina sequencing

For the methyl-CpG-binding domain sequencing (MBD-seq) analysis, 300 ng of DNA were fragmented to 200–550 bp using the methylation-insensitive restriction enzyme *MseI* (New England Biolabs, United States), which recognizes genomic T↓TAA sites, typically found outside of CGIs. The enzyme action and inactivation temperatures, as well as its actuation times and concentration were fixed according to manufacturer’s indications. Products were obtained using AMPure beads and checked and quantified with Picogreen (Invitrogen, Carlsbad, United Stated). Fragmented DNA was then submitted to methylation enrichment using the MethylCollector™ Ultra kit (Active Motif, Carlsbad, CA, United States), following the instructions. Briefly, methylated DNA was captured from 75 ng of fragmented DNA via binding to the methyl-CpG binding domain of the MBD2 protein. Illumina MBD-seq libraries were prepared from 15 ng of methylated DNA fragments using the NEBNext® Ultra™ II DNA Library Prep Kit (Illumina Inc. San Diego, CA, USA) according to the manufacturer’s instructions. All libraries were sequenced on an Illumina NovaSeq 6000 sequencer as a 1 × 75 nucleotides SE read format, according to the manufacturer’s protocol. Raw sequenced data were deposited in the Sequence Read Archive (SRA) of the National Center for Biotechnology Information (NCBI) under the Bioproject accession number PRJNA915228 (BioSample accession numbers: SAMN32381315-SAMN32381338).

Illumina RNA-seq libraries were prepared from 500 ng total RNA using the Illumina TruSeq™ Stranded mRNA LT Sample Prep Kit (Illumina Inc. San Diego, CA, USA) according to the manufacturer’s instructions. All RNA-seq libraries were sequenced on an Illumina NovaSeq 6000 sequencer as 2 × 150 nucleotides paired-end (PE) read format according to the manufacturer’s protocol. Raw sequenced data were deposited in the Sequence Read Archive (SRA) of the National Center for Biotechnology Information (NCBI) under the Bioproject accession number PRJNA915228 (BioSample accession numbers: SAMN32381339-SAMN32381362).

### Bioinformatics analyses

After sequencing, the quality of the RNA-seq and MBD-seq resulting raw reads was evaluated with FASTQC (https://www.bioinformatics.babraham.ac.uk/projects/fastqc/ accessed on 16 April 2020). In the case of RNA-seq data, libraries were filtered with Trimmomatic (RRID:SCR_011848) [90] eliminating those with quality < 18, length < 200 bp, and > 5% of Ns in the sequence. Cleaned reads were mapped against gilthead sea bream reference genome [91] (available at https://seabreamdb.nutrigroup-iats.org/), using STAR (RRID:SCR_004463) [92]. Unique transcript hit counts were calculated by using featureCounts from the Subread package [93].

In the case of MBD-seq data, pre-processing was performed with Prinseq (RRID:SCR_005454) [94], eliminating those with quality < 26, length < 60 bp, and > 5% of Ns in the sequence. Before mapping, the repeat regions of the gilthead sea bream reference were masked using the *BSgenome* R package. Then, high-quality reads were aligned to this masked genome using the Bowtie2 software (RRID:SCR_016368) [95]. After MBD-seq mapping, the methylated reads were located in their corresponding genomic region using five training sets created *ad-hoc* for this analysis. The exon and intron training sets contained the coordinates of these elements along the genome. The promoter region of a gene was considered 5,000 bp before its start codon. Finally, two training sets were established for CGI, depending on if these islands were found in promoters or intergenic regions. Predictions of CGI were done with NewCpGReport tool from the EMBOSS suite [96]. Search parameters for CGI were: length ≥ 200, C + G content ≥ 50%, ratio of observed/expected CpGs ≥ 0.60 and window size = 100.

### Statistical analysis

Data of fish performance were analysed using the analysis of variance (ANOVA) at a significance level of 0.05. Two-way ANOVA was applied to these results to determine the combined effects of genotype (GS or REF), diet (FUTURE or CTRL) and their interaction, using the program IBM SPSS version 20 for Windows (IBM SPSS Inc., Armonk, NY, USA).

DE transcripts in RNA-seq data were retrieved using DESeq2 at two significance thresholds (*P* < 0.05 and FDR < 0.05) [97]. DM regions in MBD-seq data were obtained using the *MEDIPS* R package (*P* < 0.05) [98] over regions of 25-bp size, containing at least one dinucleotide CG. To study the separation of the experimental groups, we performed several partial least-squares discriminant analysis (PLS-DA) using EZinfo v3.0 (Umetrics, Umeå, Sweden). DE transcripts and DM regions with a *P* < 0.05 were introduced in the analyses. The fitness and predictability of these models were validated by a 500 random permutation test (pR2Y < 0.05; pQ2 < 0.05) using the *ropls* R package [99]. The discriminant ability of each marker was ranked after the creation of the models according to its Variable Importance in the Projection (VIP), and useful markers were detected under a VIP ≥ 1 [100]. Over-representation tests of GO-BP terms and TFBS were implemented in the *goseq* R package [101] and statistical significance was accepted at FDR < 0.05. GO-BP levels and supra-categories were retrieved using GOATools [102]. All the networks in this work were performed with Cytoscape (RRID:SCR_003032) v2.8 [103]. Pearson correlation coefficients between the expression and methylation Log_2_FC were performed using the SigmaPlot v14.5 (Systat Software Inc.) software. Gene structure representations were obtained using the *genemodel* R package (https://github.com/greymonroe/genemodel) and the IBS software [104].

### Supplementary Information

**Additional file 1: Supplementary Table 1. **Detailed sequencing data obtained in this study.

**Additional file 2: Supplementary Table 2. **Comparison between RNA-seq results and real-time PCR validation.

**Additional file 3: Supplementary Figure 1. **Validation plots of the PLS-DA models, consisting in 500 random permutations, of RNA-seq data for GS (A) and REF (B) animals, and of MBD seq for the 4 experimental groups (C). Heatmap showing the abundance distribution (z-score) of the DE transcripts identified to be driving the separation between dietary groups in GS (D) and REF (E) fish, and heatmap showing the abundance distribution (z-score) of the DM regions identified to be driving the separation between experimental groups (C).

**Additional file 4: Supplementary Table 3A. **List of enriched GO-BP obtained from selected DE transcripts (*p* < 0.05 and VIP ≥ 1) from the PLS-DA using RNAseq data in GS animals.

**Additional file 5: Supplementary Figure 2. **Bar chart representing the distribution of DM regions in different genomic areas (intron, exon, promoter and CGI in promoter) within the transcripts showing opposite effects in DNA methylation of their DM regions and their expression in GS (A) and REF (B) fish.

**Additional file 6: Supplementary Table 4. **List and annotation of selected DM and discriminant (*p* < 0.05 and VIP ≥ 1) regions from the 5 supra-categories (classified based on the enriched GO-BP terms) with greater number of genes enriched only in GS fish.

**Additional file 7: Supplementary Figure 3. **Organization of the 23 genes selected within the Lipid metabolic process GO-BP term as potential candidate epigenetic markers.

**Additional file 8: Supplementary Table 5.** Ingredients and proximate composition of the of the gilthead sea bream broodstock diet.

## Data Availability

Most data generated or analysed during this study are included in this article and its supplementary information files. Raw sequenced data were deposited in the Sequence Read Archive (SRA) of the National Center for Biotechnology Information (NCBI) under the Bioproject accession numbers PRJNA915228 (BioSample accession numbers: SAMN32381315-SAMN32381338) and PRJNA915228 (BioSample accession numbers: SAMN32381339-SAMN32381362).

## Abbreviations

ALA: α-linolenic acid
ANOVA: Analysis of variance
AP-1: Transforming growth factor-beta 1
BW: Body weight
CGI: CpG island
CTRL: Control diet
DE: Differentially expressed
DGI: Daily growth index
DHA: Docosahexaenoic acid
DM: Differentially methylated
EPA: Eicosapentaenoic acid
EBV: Estimated breeding values
FCR: Feed conversion ratio
FUTURE: FUTURE diet
GS: Genetically selected
GO-BP: Gene ontology Biological process
HNF-3: Hepatocyte nuclear factor 3-beta
K: Condition factor
LC-PUFA: Long-chain polyunsaturated fatty acids
MBD-seq: Methyl-binding domain sequencing
NCBI: National Center for Biotechnology Information
PE: Paired-end
PLS-DA: Partial least-squares discriminant analysis
PUFA: Polyunsaturated fatty acids
REF: Reference
RINs: RNA integrity numbers
RNA-seq: Massive sequencing of mRNA
SE: Single-end
SGR: Specific growth rate
SL: Standard length
SRA: Sequence Read Archive
TFA: Total fatty acids
TFBS: Transcription factor binding sites
UD: Unique description
VIP: Variable importance in the projection
WGBS: Whole genome bisulphite sequencing
