# Supplementary material for: Broodstock nutritional programming differentially affects the hepatic transcriptome and genome-wide DNA methylome of farmed gilthead sea bream (Sparus aurata) depending on genetic background
Source: BMC Genomics. 2023 Nov 7;24:670. doi: 10.1186/s12864-023-09759-7 (PMC10631108; doi:10.1186/s12864-023-09759-7)
Supplement: Supplementary file 1 — Additional file 1: Supplementary Table 1. Detailed sequencing data obtained in this study. [file 12864_2023_9759_MOESM1_ESM.docx]

**Additional file 1: Supplementary Table 1**. Detailed sequencing data obtained in this study.

| **Experiment** | **Sample ID** | **Raw Reads** | **Pre-Processed Reads** | **Mapped Reads** | **Saturation** |
| --- | --- | --- | --- | --- | --- |
| MDB-seq | DNA_GS_CTRL_1 | 34570078 | 32627952 | 28712598 | 0.98 |
|  | DNA_GS_CTRL_2 | 42924157 | 40564668 | 35696908 | 0.99 |
|  | DNA_GS_CTRL_3 | 23857095 | 22490380 | 19791534 | 0.98 |
|  | DNA_GS_CTRL_4 | 29563789 | 27905848 | 24557146 | 0.98 |
|  | DNA_GS_CTRL_5 | 28861889 | 27206433 | 23941661 | 0.98 |
|  | DNA_GS_CTRL_6 | 26225810 | 24736646 | 21768248 | 0.98 |
|  | DNA_GS_FUTURE_1 | 29000713 | 27410993 | 24121674 | 0.99 |
|  | DNA_GS_FUTURE_2 | 40080885 | 37882369 | 33336485 | 0.98 |
|  | DNA_GS_FUTURE_3 | 32466778 | 30674751 | 26993781 | 0.98 |
|  | DNA_GS_FUTURE_4 | 27465944 | 26014318 | 22892600 | 0.98 |
|  | DNA_GS_FUTURE_5 | 25939550 | 24450160 | 21516141 | 0.98 |
|  | DNA_GS_FUTURE_6 | 30516693 | 28840975 | 25380058 | 0.98 |
|  | DNA_REF_CTRL_1 | 34275653 | 32400044 | 28512039 | 0.98 |
|  | DNA_REF_CTRL_2 | 30878841 | 29161174 | 25661833 | 0.98 |
|  | DNA_REF_CTRL_3 | 27991336 | 26428942 | 23257469 | 0.98 |
|  | DNA_REF_CTRL_4 | 29453570 | 27815532 | 24477668 | 0.98 |
|  | DNA_REF_CTRL_5 | 31471295 | 29693826 | 26130567 | 0.98 |
|  | DNA_REF_CTRL_6 | 32187770 | 30347883 | 26706137 | 0.98 |
|  | DNA_REF_FUTURE_1 | 29173488 | 27584886 | 24274700 | 0.98 |
|  | DNA_REF_FUTURE_2 | 30237455 | 28594152 | 25162854 | 0.98 |
|  | DNA_REF_FUTURE_3 | 35502707 | 33555869 | 29529165 | 0.98 |
|  | DNA_REF_FUTURE_4 | 34466509 | 32662377 | 28742892 | 0.98 |
|  | DNA_REF_FUTURE_5 | 29681984 | 28048348 | 24682546 | 0.97 |
|  | DNA_REF_FUTURE_6 | 33998935 | 32153826 | 28295367 | 0.98 |
|  |  |  |  |  |  |
| RNA-seq | RNA_GS_CTRL_1 | 54620733 | 53725635 | 49659114 |  |
|  | RNA_GS_CTRL_2 | 87639111 | 86360405 | 60166187 |  |
|  | RNA_GS_CTRL_3 | 66968171 | 65761470 | 61295543 |  |
|  | RNA_GS_CTRL_4 | 69878494 | 68721402 | 64128399 |  |
|  | RNA_GS_CTRL_5 | 61763519 | 60696994 | 56292193 |  |
|  | RNA_GS_CTRL_6 | 77844657 | 76339518 | 69896204 |  |
|  | RNA_GS_FUTURE_1 | 77177667 | 75630578 | 70421215 |  |
|  | RNA_GS_FUTURE_2 | 98162244 | 96351158 | 87944679 |  |
|  | RNA_GS_FUTURE_3 | 56871479 | 56010585 | 51804379 |  |
|  | RNA_GS_FUTURE_4 | 92592237 | 91311746 | 80490670 |  |
|  | RNA_GS_FUTURE_5 | 75669239 | 74392004 | 68300630 |  |
|  | RNA_GS_FUTURE_6 | 85419168 | 84097003 | 81111438 |  |
|  | RNA_REF_CTRL_1 | 104074023 | 102148065 | 96109853 |  |
|  | RNA_REF_CTRL_2 | 61451337 | 60400405 | 55921130 |  |
|  | RNA_REF_CTRL_3 | 74414119 | 72937081 | 68067673 |  |
|  | RNA_REF_CTRL_4 | 90866350 | 89323445 | 78903494 |  |
|  | RNA_REF_CTRL_5 | 120460267 | 118338949 | 108142773 |  |
|  | RNA_REF_CTRL_6 | 114564866 | 112753821 | 102186115 |  |
|  | RNA_REF_FUTURE_1 | 106448361 | 104272368 | 96584415 |  |
|  | RNA_REF_FUTURE_2 | 75701723 | 74250739 | 69951281 |  |
|  | RNA_REF_FUTURE_3 | 71181834 | 69904162 | 65338746 |  |
|  | RNA_REF_FUTURE_4 | 75773099 | 74470126 | 70676110 |  |
|  | RNA_REF_FUTURE_5 | 92091834 | 90565740 | 85155373 |  |
|  | RNA_REF_FUTURE_6 | 117121332 | 114802678 | 101488700 |  |
