# Supplementary material for: Broodstock nutritional programming differentially affects the hepatic transcriptome and genome-wide DNA methylome of farmed gilthead sea bream (Sparus aurata) depending on genetic background
Source: BMC Genomics. 2023 Nov 7;24:670. doi: 10.1186/s12864-023-09759-7 (PMC10631108; doi:10.1186/s12864-023-09759-7)
Supplement: Supplementary file 2 — Additional file 2: Supplementary Table 2. Comparison between RNA-seq results and real-time PCR validation. [file 12864_2023_9759_MOESM2_ESM.docx]

Additional file 2: Supplementary Table 2. Comparison between RNA-seq results and real-time PCR validation.

|  |  |  | **Fold-change (FUTURE vs CTRL)** | |
| --- | --- | --- | --- | --- |
| **Gene Name** | **Symbol** | **Genbank Accession** | **RNA-seq** | **Real-time PCR** |
| Fatty acid synthase | *fasn* | JQ277708 | -4.15 | -3.86 |
| Acyl-CoA desaturase | *scd1a* | JQ277703 | -2.52 | -2.26 |
| Mitofusin-1 | *mfn1* | JX975250 | -1.20 | -1.29 |
| Peroxisome proliferator-activated receptor alpha | *pparα* | AY590299 | 1.54 | 1.76 |
| Lipoprotein lipase-like | *lpl-like* | JQ390609 | 3.22 | 2.96 |
| Platelet glycoprotein 4 | *cd36* | XM_030440140 | 15.79 | 16.01 |
