# Supplementary figures and images for: Broodstock nutritional programming differentially affects the hepatic transcriptome and genome-wide DNA methylome of farmed gilthead sea bream (Sparus aurata) depending on genetic background
Source: BMC Genomics. 2023 Nov 7;24:670. doi: 10.1186/s12864-023-09759-7 (PMC10631108; doi:10.1186/s12864-023-09759-7)

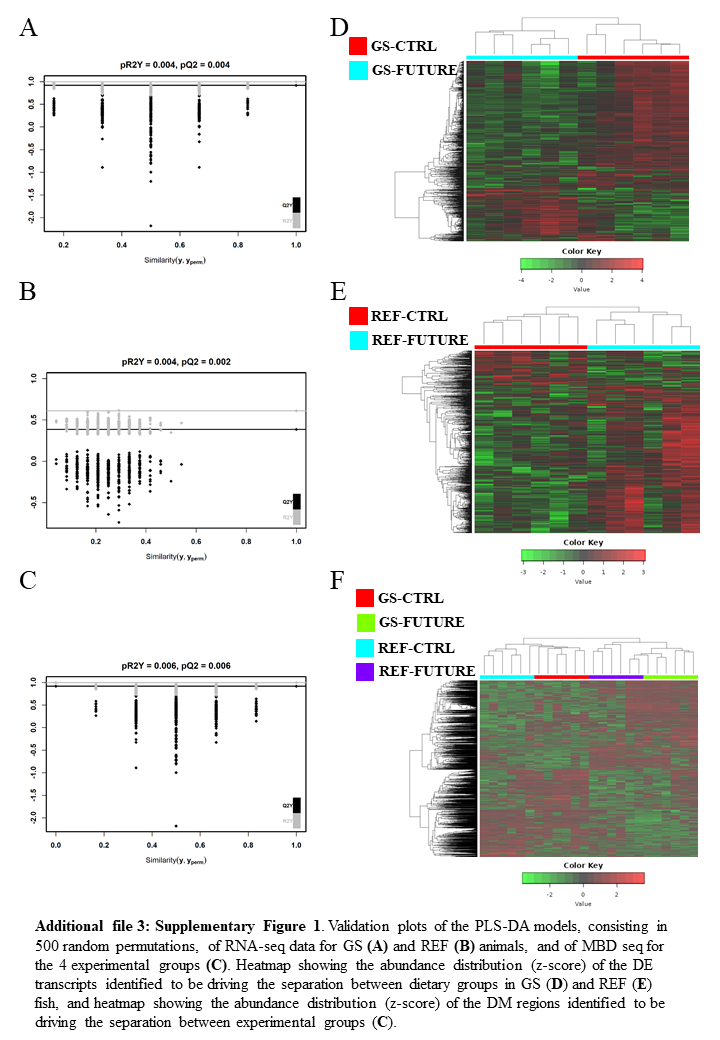

Supplement: Supplementary file 3 — Additional file 3: Supplementary Figure 1. Validation plots of the PLS-DA models, consisting in 500 random permutations, of RNA-seq data for GS (A) and REF (B) animals, and of MBD seq for the 4 experimental groups (C). Heatmap showing the abundance distribution (z-score) of the DE transcripts identified to be driving the separation between dietary groups in GS (D) and REF (E) fish, and heatmap showing the abundance distribution (z-score) of the DM regions identified to be driving the separation between experimental groups (C). [file 12864_2023_9759_MOESM3_ESM.tif]

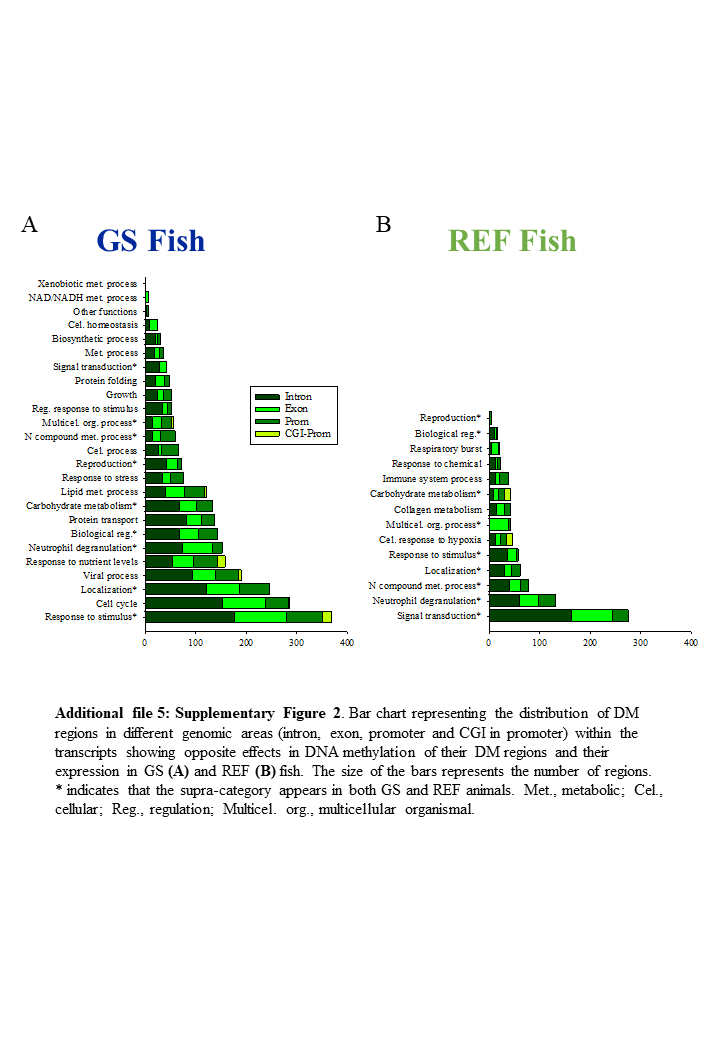

Supplement: Supplementary file 5 — Additional file 5: Supplementary Figure 2. Bar chart representing the distribution of DM regions in different genomic areas (intron, exon, promoter and CGI in promoter) within the transcripts showing opposite effects in DNA methylation of their DM regions and their expression in GS (A) and REF (B) fish. [file 12864_2023_9759_MOESM5_ESM.tif]
