# Supplementary material for: Broodstock nutritional programming differentially affects the hepatic transcriptome and genome-wide DNA methylome of farmed gilthead sea bream (Sparus aurata) depending on genetic background
Source: BMC Genomics. 2023 Nov 7;24:670. doi: 10.1186/s12864-023-09759-7 (PMC10631108; doi:10.1186/s12864-023-09759-7)
Supplement: Supplementary file 7 — Additional file 7: Supplementary Figure 3. Organization of the 23 genes selected within the Lipid metabolic process GO-BP term as potential candidate epigenetic markers. [file 12864_2023_9759_MOESM7_ESM.pptx]

## Slide 1
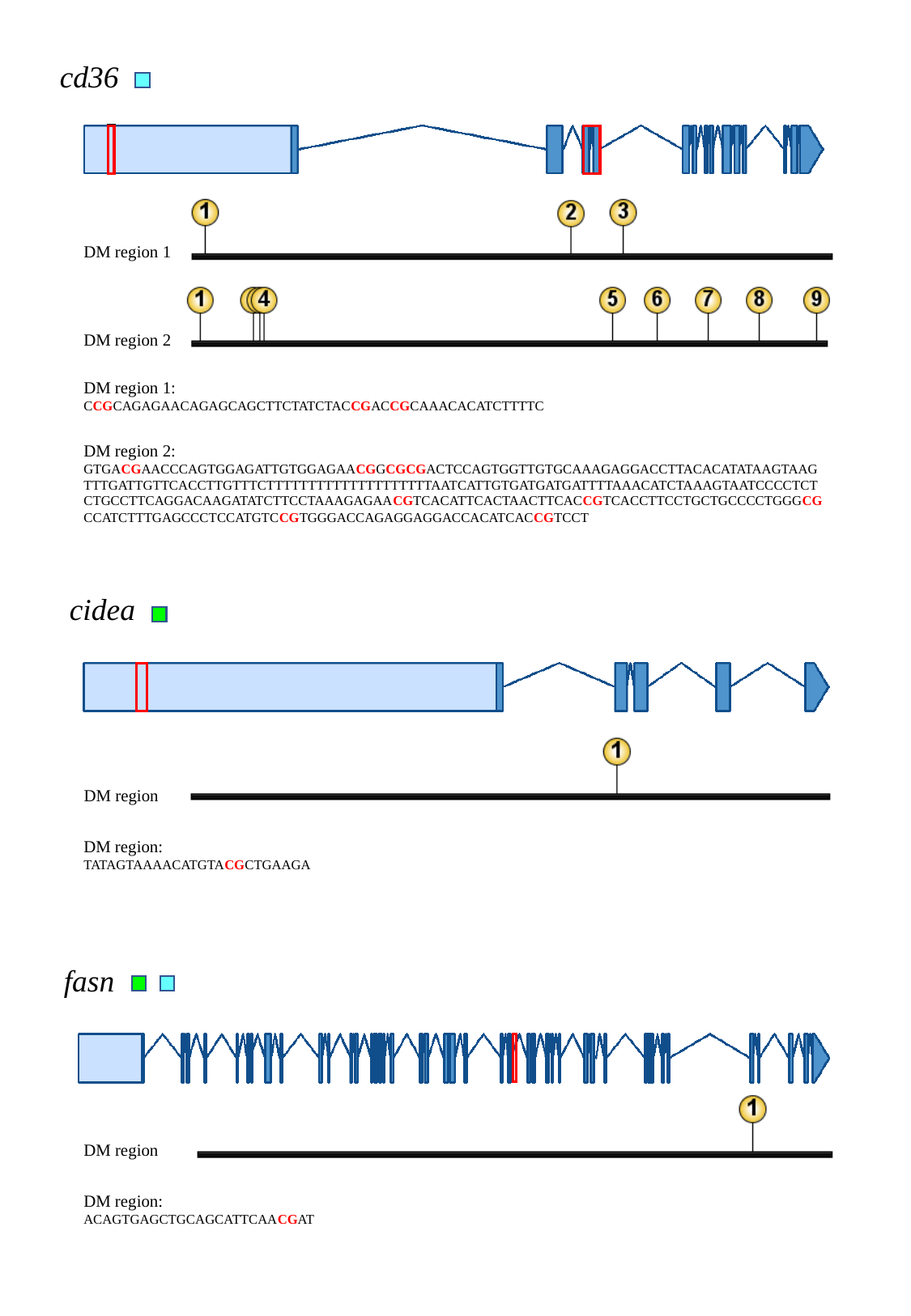

cd36
DM region 1
DM region 2
DM region 1:
CCGCAGAGAACAGAGCAGCTTCTATCTACCGACCGCAAACACATCTTTTC
DM region 2: GTGACGAACCCAGTGGAGATTGTGGAGAACGGCGCGACTCCAGTGGTTGTGCAAAGAGGACCTTACACATATAAGTAAGTTTGATTGTTCACCTTGTTTCTTTTTTTTTTTTTTTTTTTTAATCATTGTGATGATGATTTTAAACATCTAAAGTAATCCCCTCTCTGCCTTCAGGACAAGATATCTTCCTAAAGAGAACGTCACATTCACTAACTTCACCGTCACCTTCCTGCTGCCCCTGGGCGCCATCTTTGAGCCCTCCATGTCCGTGGGACCAGAGGAGGACCACATCACCGTCCT
cidea
DM region
DM region:
TATAGTAAAACATGTACGCTGAAGA
fasn
DM region
DM region:
ACAGTGAGCTGCAGCATTCAACGAT

## Slide 2
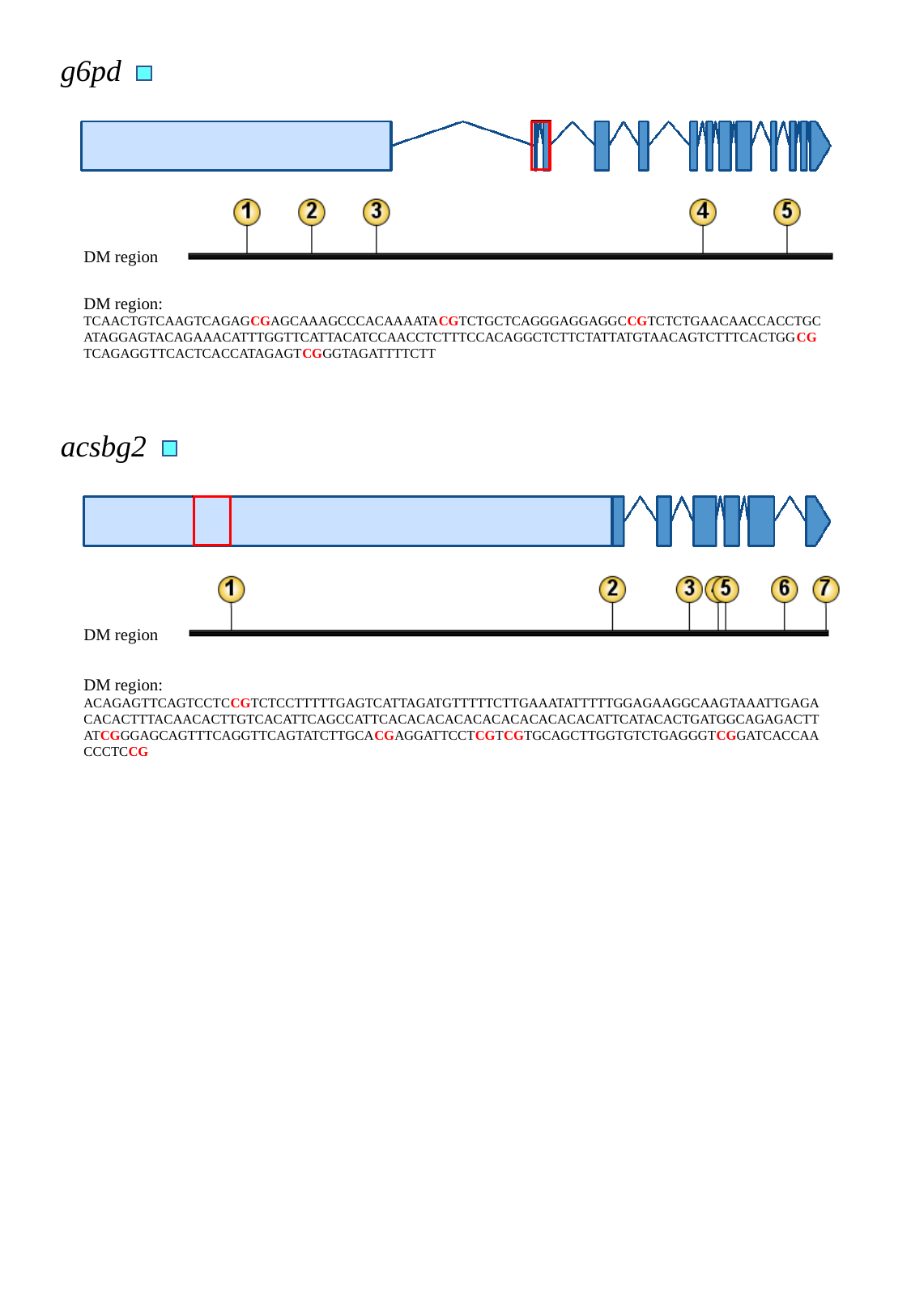

g6pd
DM region
DM region: TCAACTGTCAAGTCAGAGCGAGCAAAGCCCACAAAATACGTCTGCTCAGGGAGGAGGCCGTCTCTGAACAACCACCTGCATAGGAGTACAGAAACATTTGGTTCATTACATCCAACCTCTTTCCACAGGCTCTTCTATTATGTAACAGTCTTTCACTGGCGTCAGAGGTTCACTCACCATAGAGTCGGGTAGATTTTCTT
acsbg2
DM region
DM region: ACAGAGTTCAGTCCTCCGTCTCCTTTTTGAGTCATTAGATGTTTTTCTTGAAATATTTTTGGAGAAGGCAAGTAAATTGAGACACACTTTACAACACTTGTCACATTCAGCCATTCACACACACACACACACACACACATTCATACACTGATGGCAGAGACTTATCGGGAGCAGTTTCAGGTTCAGTATCTTGCACGAGGATTCCTCGTCGTGCAGCTTGGTGTCTGAGGGTCGGATCACCAACCCTCCG

## Slide 3
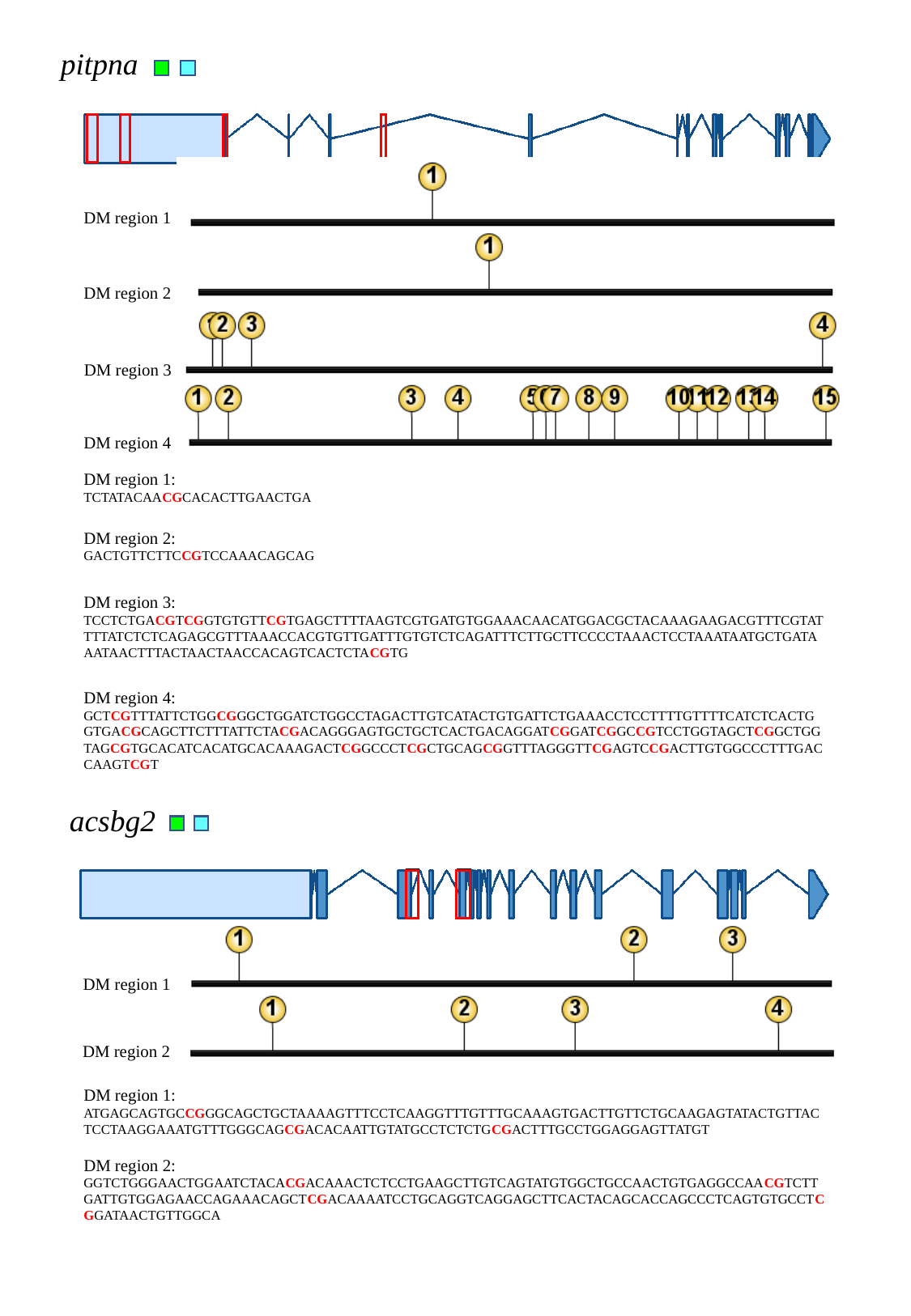

pitpna
DM region 1
DM region 2
DM region 3
DM region 4
DM region 1:
TCTATACAACGCACACTTGAACTGA
DM region 2:
GACTGTTCTTCCGTCCAAACAGCAG
DM region 3: TCCTCTGACGTCGGTGTGTTCGTGAGCTTTTAAGTCGTGATGTGGAAACAACATGGACGCTACAAAGAAGACGTTTCGTATTTTATCTCTCAGAGCGTTTAAACCACGTGTTGATTTGTGTCTCAGATTTCTTGCTTCCCCTAAACTCCTAAATAATGCTGATAAATAACTTTACTAACTAACCACAGTCACTCTACGTG
DM region 4: GCTCGTTTATTCTGGCGGGCTGGATCTGGCCTAGACTTGTCATACTGTGATTCTGAAACCTCCTTTTGTTTTCATCTCACTGGTGACGCAGCTTCTTTATTCTACGACAGGGAGTGCTGCTCACTGACAGGATCGGATCGGCCGTCCTGGTAGCTCGGCTGGTAGCGTGCACATCACATGCACAAAGACTCGGCCCTCGCTGCAGCGGTTTAGGGTTCGAGTCCGACTTGTGGCCCTTTGACCAAGTCGT
acsbg2
DM region 1
DM region 2
DM region 1: ATGAGCAGTGCCGGGCAGCTGCTAAAAGTTTCCTCAAGGTTTGTTTGCAAAGTGACTTGTTCTGCAAGAGTATACTGTTACTCCTAAGGAAATGTTTGGGCAGCGACACAATTGTATGCCTCTCTGCGACTTTGCCTGGAGGAGTTATGT
DM region 2: GGTCTGGGAACTGGAATCTACACGACAAACTCTCCTGAAGCTTGTCAGTATGTGGCTGCCAACTGTGAGGCCAACGTCTTGATTGTGGAGAACCAGAAACAGCTCGACAAAATCCTGCAGGTCAGGAGCTTCACTACAGCACCAGCCCTCAGTGTGCCTCGGATAACTGTTGGCA

## Slide 4
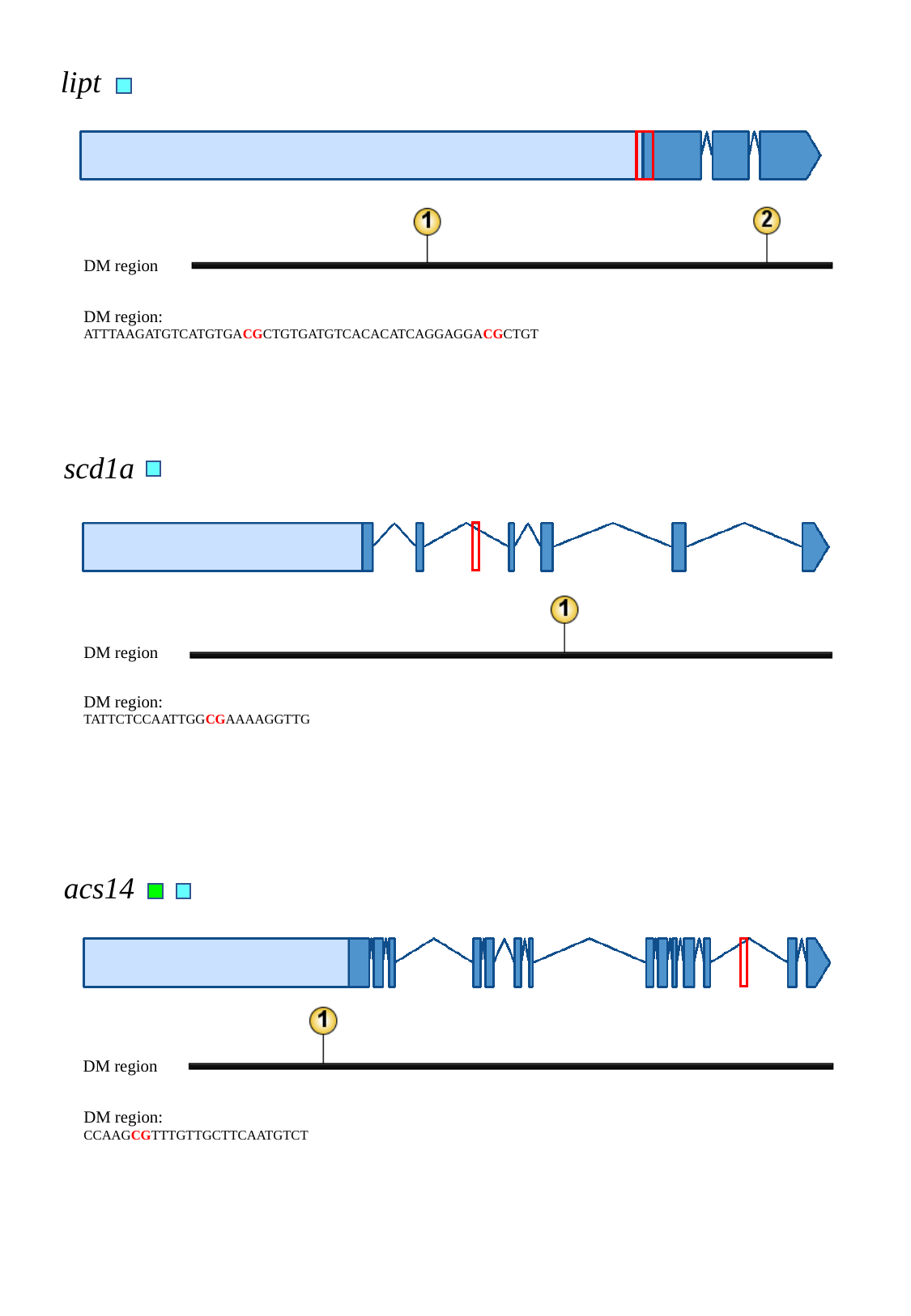

lipt
DM region
DM region:
ATTTAAGATGTCATGTGACGCTGTGATGTCACACATCAGGAGGACGCTGT
scd1a
DM region
DM region:
TATTCTCCAATTGGCGAAAAGGTTG
acs14
DM region
DM region:
CCAAGCGTTTGTTGCTTCAATGTCT

## Slide 5
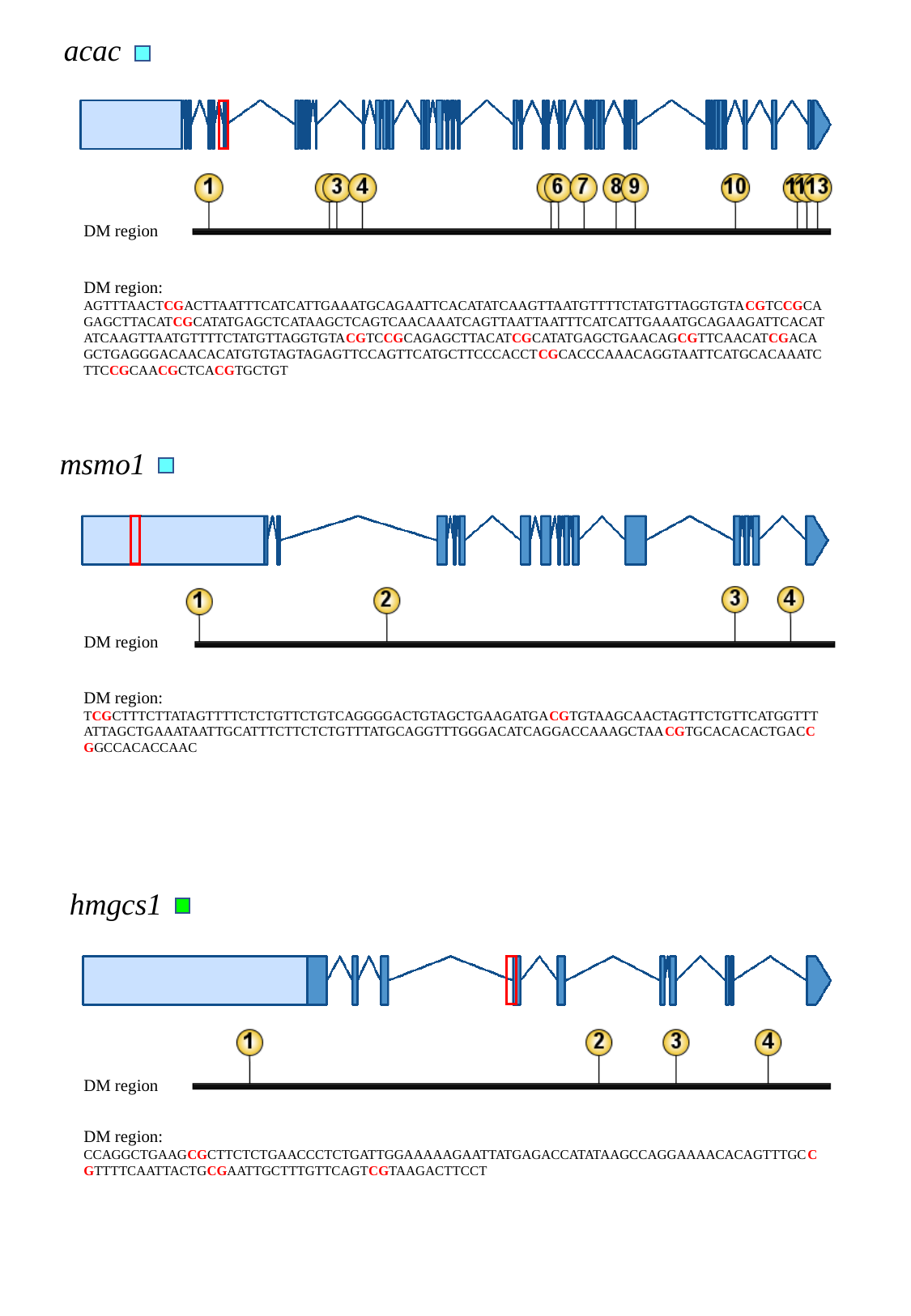

acac
DM region
DM region:
AGTTTAACTCGACTTAATTTCATCATTGAAATGCAGAATTCACATATCAAGTTAATGTTTTCTATGTTAGGTGTACGTCCGCAGAGCTTACATCGCATATGAGCTCATAAGCTCAGTCAACAAATCAGTTAATTAATTTCATCATTGAAATGCAGAAGATTCACATATCAAGTTAATGTTTTCTATGTTAGGTGTACGTCCGCAGAGCTTACATCGCATATGAGCTGAACAGCGTTCAACATCGACAGCTGAGGGACAACACATGTGTAGTAGAGTTCCAGTTCATGCTTCCCACCTCGCACCCAAACAGGTAATTCATGCACAAATCTTCCGCAACGCTCACGTGCTGT
msmo1
DM region
DM region: TCGCTTTCTTATAGTTTTCTCTGTTCTGTCAGGGGACTGTAGCTGAAGATGACGTGTAAGCAACTAGTTCTGTTCATGGTTTATTAGCTGAAATAATTGCATTTCTTCTCTGTTTATGCAGGTTTGGGACATCAGGACCAAAGCTAACGTGCACACACTGACCGGCCACACCAAC
hmgcs1
DM region
DM region: CCAGGCTGAAGCGCTTCTCTGAACCCTCTGATTGGAAAAAGAATTATGAGACCATATAAGCCAGGAAAACACAGTTTGCCGTTTTCAATTACTGCGAATTGCTTTGTTCAGTCGTAAGACTTCCT

## Slide 6
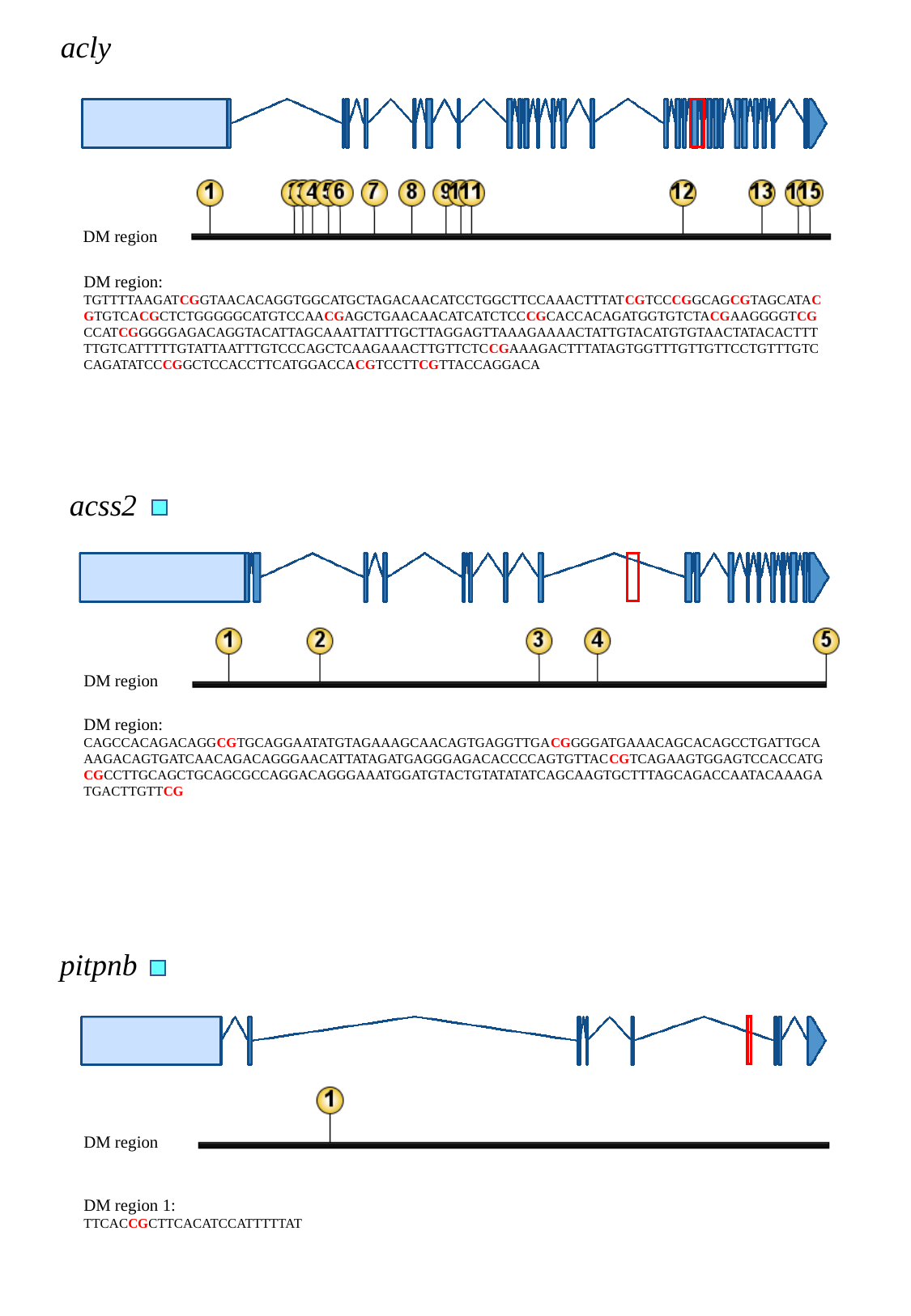

acly
DM region
DM region: TGTTTTAAGATCGGTAACACAGGTGGCATGCTAGACAACATCCTGGCTTCCAAACTTTATCGTCCCGGCAGCGTAGCATACGTGTCACGCTCTGGGGGCATGTCCAACGAGCTGAACAACATCATCTCCCGCACCACAGATGGTGTCTACGAAGGGGTCGCCATCGGGGGAGACAGGTACATTAGCAAATTATTTGCTTAGGAGTTAAAGAAAACTATTGTACATGTGTAACTATACACTTTTTGTCATTTTTGTATTAATTTGTCCCAGCTCAAGAAACTTGTTCTCCGAAAGACTTTATAGTGGTTTGTTGTTCCTGTTTGTCCAGATATCCCGGCTCCACCTTCATGGACCACGTCCTTCGTTACCAGGACA
acss2
DM region
DM region: CAGCCACAGACAGGCGTGCAGGAATATGTAGAAAGCAACAGTGAGGTTGACGGGGATGAAACAGCACAGCCTGATTGCAAAGACAGTGATCAACAGACAGGGAACATTATAGATGAGGGAGACACCCCAGTGTTACCGTCAGAAGTGGAGTCCACCATGCGCCTTGCAGCTGCAGCGCCAGGACAGGGAAATGGATGTACTGTATATATCAGCAAGTGCTTTAGCAGACCAATACAAAGATGACTTGTTCG
pitpnb
DM region
DM region 1:
TTCACCGCTTCACATCCATTTTTAT

## Slide 7
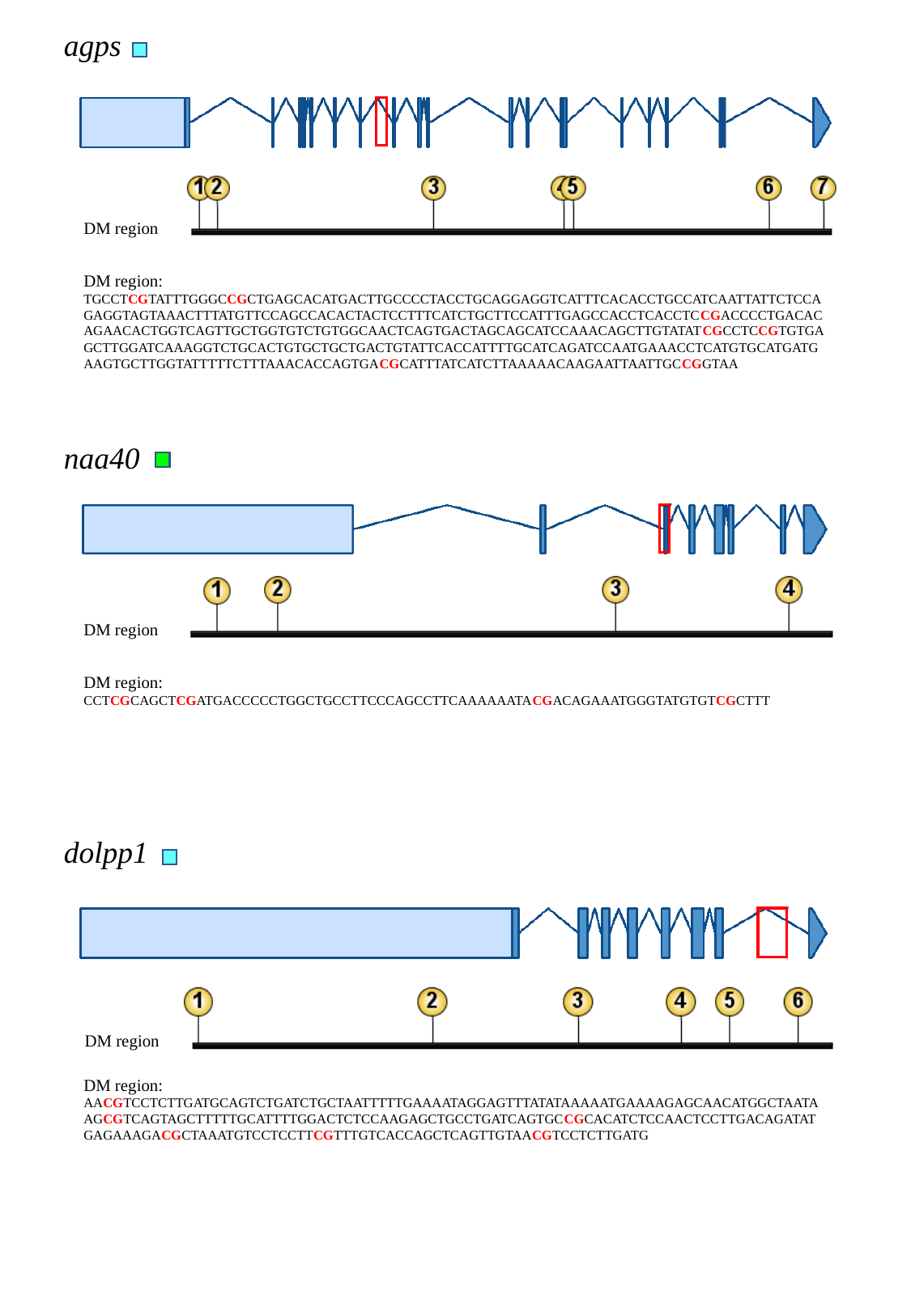

agps
DM region
DM region: TGCCTCGTATTTGGGCCGCTGAGCACATGACTTGCCCCTACCTGCAGGAGGTCATTTCACACCTGCCATCAATTATTCTCCAGAGGTAGTAAACTTTATGTTCCAGCCACACTACTCCTTTCATCTGCTTCCATTTGAGCCACCTCACCTCCGACCCCTGACACAGAACACTGGTCAGTTGCTGGTGTCTGTGGCAACTCAGTGACTAGCAGCATCCAAACAGCTTGTATATCGCCTCCGTGTGAGCTTGGATCAAAGGTCTGCACTGTGCTGCTGACTGTATTCACCATTTTGCATCAGATCCAATGAAACCTCATGTGCATGATGAAGTGCTTGGTATTTTTCTTTAAACACCAGTGACGCATTTATCATCTTAAAAACAAGAATTAATTGCCGGTAA
naa40
DM region
DM region: CCTCGCAGCTCGATGACCCCCTGGCTGCCTTCCCAGCCTTCAAAAAATACGACAGAAATGGGTATGTGTCGCTTT
dolpp1
DM region
DM region: AACGTCCTCTTGATGCAGTCTGATCTGCTAATTTTTGAAAATAGGAGTTTATATAAAAATGAAAAGAGCAACATGGCTAATAAGCGTCAGTAGCTTTTTGCATTTTGGACTCTCCAAGAGCTGCCTGATCAGTGCCGCACATCTCCAACTCCTTGACAGATATGAGAAAGACGCTAAATGTCCTCCTTCGTTTGTCACCAGCTCAGTTGTAACGTCCTCTTGATG

## Slide 8
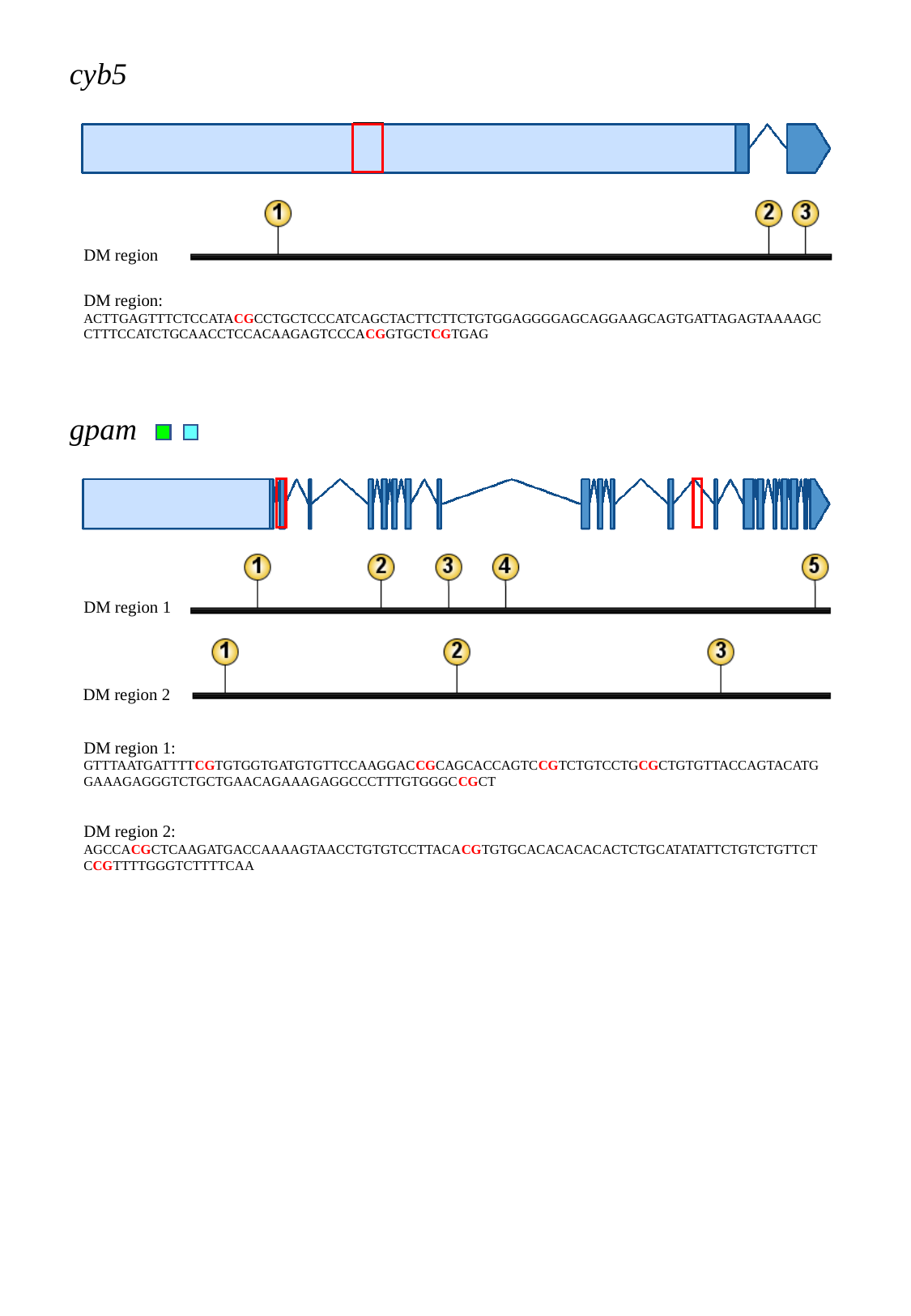

cyb5
DM region
DM region: ACTTGAGTTTCTCCATACGCCTGCTCCCATCAGCTACTTCTTCTGTGGAGGGGAGCAGGAAGCAGTGATTAGAGTAAAAGCCTTTCCATCTGCAACCTCCACAAGAGTCCCACGGTGCTCGTGAG
gpam
DM region 1
DM region 2
DM region 1: GTTTAATGATTTTCGTGTGGTGATGTGTTCCAAGGACCGCAGCACCAGTCCGTCTGTCCTGCGCTGTGTTACCAGTACATGGAAAGAGGGTCTGCTGAACAGAAAGAGGCCCTTTGTGGGCCGCT
DM region 2: AGCCACGCTCAAGATGACCAAAAGTAACCTGTGTCCTTACACGTGTGCACACACACACTCTGCATATATTCTGTCTGTTCTCCGTTTTGGGTCTTTTCAA

## Slide 9
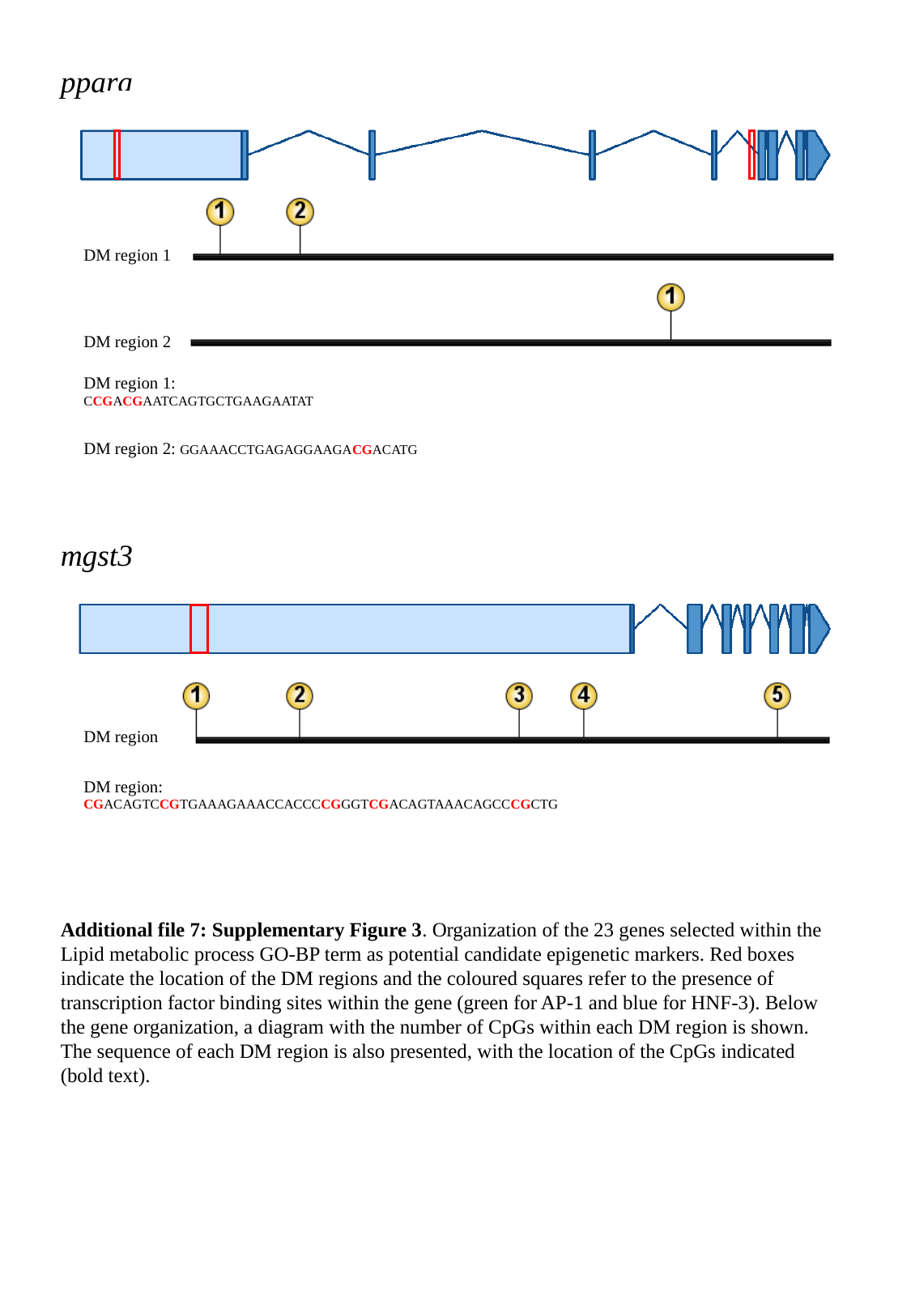

ppara
DM region 1
DM region 2
DM region 1:
CCGACGAATCAGTGCTGAAGAATAT
DM region 2: GGAAACCTGAGAGGAAGACGACATG
mgst3
DM region
DM region:
CGACAGTCCGTGAAAGAAACCACCCCGGGTCGACAGTAAACAGCCCGCTG
Additional file 7: Supplementary Figure 3. Organization of the 23 genes selected within the Lipid metabolic process GO-BP term as potential candidate epigenetic markers. Red boxes indicate the location of the DM regions and the coloured squares refer to the presence of transcription factor binding sites within the gene (green for AP-1 and blue for HNF-3). Below the gene organization, a diagram with the number of CpGs within each DM region is shown. The sequence of each DM region is also presented, with the location of the CpGs indicated (bold text).
