# Supplementary material for: Broodstock nutritional programming differentially affects the hepatic transcriptome and genome-wide DNA methylome of farmed gilthead sea bream (Sparus aurata) depending on genetic background
Source: BMC Genomics. 2023 Nov 7;24:670. doi: 10.1186/s12864-023-09759-7 (PMC10631108; doi:10.1186/s12864-023-09759-7)
Supplement: Supplementary file 8 — Additional file 8: Supplementary Table 5. Ingredients and proximate composition of the of the gilthead sea bream broodstock diet. [file 12864_2023_9759_MOESM8_ESM.docx]

**Additional file 8: Supplementary Table 5.** Ingredients and proximate composition of the of the gilthead sea bream broodstock diet.

| Ingredients (%): | | | | | | | Broodstock diet |
| --- | --- | --- | --- | --- | --- | --- | --- |
| Fish meal (North-Atlantic 12 C) | | | | | | | 59.36 |
| Krill meal | | | | | | | 7.00 |
| Squid meal | | | | | | | 3.00 |
| Wheat | | | | | | | 20.57 |
| Fish oil (South American) | | | | | | | 1.76 |
| Rapeseed oil | | | | | | | 7.54 |
| Vitamin-mineral premix | | | | | | | 0.50 |
| L-Histidine HCl | | | | | | | 0.27 |
|  |  |  |  |  |  |  |  |
| Proximate composition | | | | | | |  |
| Crude protein (% dry matter, DM) | | | | | | | 58.1 |
| Crude lipid (% DM) | | | | | | | 22.1 |
| Ash (% DM) | | | | | | | 9.8 |
